# Partial Deletion of the Carboxyl‐Terminal Signal Sequence of the Cellular Prion Protein Alters Protein Expression via Endoplasmic Reticulum–Associated Degradation

**DOI:** 10.1096/fj.202501227RR

**Published:** 2025-09-04

**Authors:** Miryeong Yoo, Sungeun Lee, Jieun Kim, Sunyeong Cha, Min Young Lee, Yeon Jeong Hwang, Woo‐Ri Ko, Taeeun Kim, A‐ran Kim, Trang H. T. Trinh, Young‐Mi Kim, Yong‐Pil Cheon, Chongsuk Ryou

**Affiliations:** ^1^ Department of Pharmacy College of Pharmacy, and Institute of Pharmaceutical Science & Technology, Hanyang University ERICA Ansan Republic of Korea; ^2^ Division of Developmental Biology and Physiology, Department of Biotechnology Institute for Basic Sciences, Sungshin University Seoul Republic of Korea; ^3^ Department of Pharmacy East Asia University of Technology Hanoi Vietnam

**Keywords:** endoplasmic reticulum–associated degradation, gene expression regulation, GPI‐linked proteins, prion proteins, unfolded protein response

## Abstract

Cellular prion protein (PrP^C^) is a glycoprotein tethered to the plasma membrane via a GPI‐anchor, and it plays a crucial role in prion diseases by undergoing conformational change to PrP^Sc^. To generate a knock‐in (KI) mouse model expressing bank vole PrP^C^ (BVPrP^C^), a KI targeting construct was designed. However, a *Prnp* gene sequence that encodes PrP^C^ lacking seven C‐terminal amino acid residues of the GPI‐anchoring signal sequence (GPI‐SS) was unintentionally introduced into the construct. The resulting KIBVPrP248 mice exhibited very low PrP^C^ expression and resistance to prion infection. To investigate the underlying mechanism of reduced PrP^C^ expression, RK13 cells expressing either full‐length GPI‐SS (BVPrP255) or truncated GPI‐SS (BVPrP248) and KIBVPrP248 mice were analyzed. In RK13‐BVPrP248 cells, PrP^C^ protein levels were nearly ten‐fold lower than in RK13‐BVPrP255 cells, mimicking the extremely low PrP^C^ expression of the KIBVPrP248 mice. The abundance, stability, and translational efficiency of the *Prnp* mRNA were not the primary causes for the low PrP^C^ expression in RK13‐BVPrP248 cells. A pharmacological analysis revealed that BVPrP248 underwent enhanced degradation via the ER‐associated degradation pathway, with increased PrP ubiquitination detected in both the cell and animal models. An immunofluorescence analysis showed that BVPrP248 was mislocalized to the ER, co‐localizing with Grp78, an ER chaperone. Although mislocalization of BVPrP248 under the transient overexpression condition led to mild activation of the unfolded protein response in RK13‐BVPrP248 cells, low‐level chronic expression of BVPrP248 in stable transfectants and KIBVPrP248 mice did not facilitate such events. These findings suggested that the C‐terminal GPI‐SS of PrP^C^ plays a critical role in PrP^C^ biogenesis.

## Graphical abstract

Knock‐in mice expressing PrP with a partial deletion of the C‐terminal signal sequence (KIBVPrP248) showed reduced PrPC expression and resistance to prion infection. The underlying mechanism is that the mutant PrP is aberrantly retained in the endoplasmic reticulum and eventually undergoes proteasomal degradation. ER, endoplasmic reticulum; UPR, unfolded protein response; PM, plasma membrane. The graphic was created using BioRender (https://www.biorender.com/).

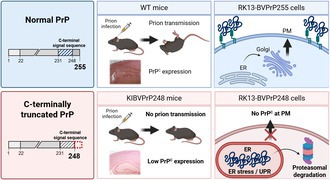

## 1 Introduction

The cellular prion protein (PrP^C^) is a glycosylphosphatidylinositol (GPI)‐anchored glycoprotein expressed on the plasma membranes of cells [1, 2]. PrP^C^ is predominantly expressed in the central nervous system and is highly conserved across various mammalian species [3, 4, 5]. During early translation, the 22 N‐terminal amino acids of PrP^C^ serve as a signal sequence that directs the mRNA‐ribosome complex to the translocation channel on the endoplasmic reticulum (ER) membrane through complexation with a signal recognition particle and its receptor [6]. The polypeptide chain is synthesized in the ER and passes through to the translocation channel. Then, the signal peptide is cleaved by a signal peptidase located on the luminal side of the ER membrane, while translation continues and the rest of PrP^C^ polypeptide is put within the ER lumen [7]. During this process, an oligosaccharyltransferase recognizes the NXT amino acid motifs in PrP^C^ as glycan attachment sites, and they subsequently undergo N‐glycosylation [8]. Additionally, a single disulfide bond forms between two cysteine residues at the C‐terminus [9]. The GPI‐anchoring signal sequence (GPI‐SS) in PrP^C^ is recognized and cleaved by the GPI transamidase complex, which replaces it with a GPI‐anchor [10]. Once the protein is transported to the Golgi apparatus, the GPI‐anchor undergoes lipid remodeling and additional carbohydrate side‐chain modifications, ultimately localizing to lipid rafts on the cell membrane through GPI‐anchoring [11]. Thus, the GPI‐anchor plays a crucial role in the proper localization of PrP^C^ to the plasma membrane.

Several different mutation types in the GPI‐SS of PrP, such as point mutations, complete or partial deletions, and replacement with corresponding sequences from other proteins, are associated with prion diseases. In mice expressing human PrP with the M232R mutation, the development of prion disease was accelerated in a strain‐dependent manner [12]. Although the protein expression and subcellular localization of PrP^C^ were similar to those in wild type (WT) mice, decreased N‐glycosylation and enhanced PrP^C^ truncation were observed. This was attributed to altered ER translocation caused by the reduced hydrophobicity of the GPI‐SS [12]. In ScN2a cells expressing mouse PrP^C^ with an amino acid substitution at codon 230 or 232, which juxtaposes the cleavage site of GPI‐SS, total PrP expression levels were reduced, and the mutant PrP was retained in the ER [13]. In studies of PrP with complete deletion of the GPI‐SS in several cell types, anchorless PrP^C^ was secreted into the extracellular space [14, 15, 16]. Mouse fibroblast cells expressing anchorless mouse PrP^C^ were reported to resist infection by the 22 L scrapie strain [17]. When transgenic mice expressing anchorless mouse PrP were infected with the RML, ME7, or 22 L prion strains, they did not exhibit clinical signs, but an accumulation of the infectious amyloid form of PrP was detected [18, 19]. Additionally, one study suggested that anchorless PrP can generate a novel form of infectious prion following RML prion infection [20], and another reported spontaneous PrP amyloid formation without prion inoculation [21]. Studies in which the GPI‐SS of PrP was replaced with that of other proteins have also provided valuable insights. When the GPI‐SS of PrP^C^ was replaced with that of Thy‐1, prion disease progression was delayed in mouse models [22], and replacement with the GPI‐SS of CD59 altered ER translocation pathways in cultured cells [23]. Collectively, these findings demonstrate that mutations affecting GPI‐anchoring have a significant effect on the subsequent maturation and subcellular localization of the protein and prion disease progression.

In our ongoing effort to establish knock‐in (KI) mouse lines expressing bank vole (BV, *Myodes glareolus* ) PrP^C^, a BVPrP sequence (GenBank: AF367624.1) missing seven amino acid residues (amino acids 249–255; LIFLIVG) in the C‐terminal GPI‐SS was unintentionally introduced to mice. The resultant mouse line showed abnormal PrP^C^ expression and resistance to prion infection, which will be described in detail in this report. Our findings suggest that the seven amino acids deleted from the C‐terminus of the BVPrP GPI‐SS play a role in PrP^C^ expression and subsequent prion propagation.

In this study, we elucidate how partial deletion of the C‐terminal GPI‐SS of PrP^C^ suppresses PrP^C^ expression at the molecular level. Using cultured cells and KI mice generated to express BVPrP^C^ lacking seven amino acids in the C‐terminal signal sequence, we investigated the transcriptional, post‐transcriptional, translational, and post‐translational processes of protein biosynthesis. Furthermore, we examined the effects of this deletion on subcellular localization, N‐linked glycosylation, and GPI‐anchoring of PrP^C^. In addition, we investigated the cellular response caused by aberrant PrP^C^ expression.

## 2 Materials and Methods

### 2.1 Generation of a BVPrP KI Mouse Line

The animal experiments were performed with the approval of the Institutional Animal Care and Use Committee at Hanyang University, Seoul, Republic of Korea (HY‐IACUC‐20‐0011, HY‐IACUC‐21‐0016, HY‐IACUC‐21‐0049, HY‐IACUC‐22‐0032, HY‐IACUC‐24‐0006). To generate BVPrP KI mice, a targeting vector was designed with homologous sequences at the 5′ and 3′ ends, allowing the BVPrP^C^ sequence to be inserted between the start and stop codons of the mouse *Prnp* gene. The middle region of the vector sequentially contained the BVPrP cDNA sequence, loxP site, poly(A) signal, neomycin resistance gene (Neo^R^), and loxP site (Figure S1A). The KI mice were generated at the transgenic facility of Cyagen (Santa Clara, CA, USA) according to the manufacturer's standard operating protocol. Briefly, the targeting vector was linearized by NotI and electroporated into embryonic stem (ES) cells obtained from C57BL/6N mice. To monitor the successful integration of the KI construct into the ES cell genome, genomic DNA from ES cells was digested with BstEII and KpnI and subjected to a Southern blot analysis using a Neo^R^‐specific probe. Among the successfully targeted ES cell clones, clone 1A8 was selected for further experiments. Cre recombinase was then introduced into the validated ES cell clone 1A8 to excise the Neo^R^ gene between the two loxP sites, generating KIBVPrP ES cells devoid of the selection cassette. The targeted ES cell clone (1A8) was subsequently microinjected into blastocysts derived from fertilized embryos of C57BL/6N albino mice. The injected blastocysts were then re‐implanted into pseudo‐pregnant CD‐1 females. Chimeric animals were identified by their coat color. Germline transmission was confirmed by breeding with C57BL/6N females and subsequent genotyping of the offspring. Three male and five female heterozygous targeted mice were generated and interbred to generate homozygous KI mice at the animal facility of the Center for Laboratory Animal Science (Hanyang University ERICA, Ansan, Korea). Offspring were genotyped, and homozygous mice were identified for further breeding to ensure stable inheritance of the targeted allele. Finally, the KI mouse line B6; B6‐*Prnp* ^*tm1Rc*^ */R* (KIBVPrP248) was established. The KI allele *Rc* (the initial of Ryou and Cheon who collaborated to establish the mouse line) indicates the targeted mutant allele encoding the BVPrP with M109 polymorphism and the deletion of amino acid residues 249 to 255 (BV*Prnp*M109, Δ249–255).

### 2.2 Genotyping

For genotyping, multiplex PCR was performed using a combination of the mouse PrP forward, BVPrP forward, and common reverse primers, together with the internal control glyceraldehyde 3‐phosphate dehydrogenase (GAPDH) forward and reverse primers (Figure S1B box, Set 1 in Table S1). The mouse forward primer was designed based on the mouse PrP cDNA sequence, and the BVPrP forward primer was designed based on the BVPrP cDNA sequence. The common reverse primer targeted the 3′ untranslated region (UTR) of exon 3 in the mouse *Prnp* gene. The mouse PrP‐specific primer set produces a PCR product of 167 bp, whereas the BVPrP‐specific primer set yields a 206‐bp PCR product for the BVPrP KI allele because it includes a loxP site between the BVPrP cDNA sequence and the 3′ UTR. The internal control GAPDH primer set produces a 500‐bp PCR product. Genomic DNA (gDNA) was extracted from the tail at 55°C overnight in a buffer containing 50 mM Tris–HCl (pH 8.0; Sigma‐Aldrich, St. Louis, MO, USA), 100 mM EDTA (pH 8.0; Sigma‐Aldrich), 100 mM NaCl (Sigma‐Aldrich), 1% sodium dodecyl sulfate (SDS; Sigma‐Aldrich), and 400 μg/mL proteinase K (PK; Roche, Basel, Switzerland). PCR conditions were as follows: 3 min at 94°C; 38 cycles of 30 s at 94°C, 35 s at 60°C, and 35 s at 72°C; followed by a final extension of 5 min at 72°C. PCR was carried out using AccuPower PCR premix (Bioneer, Daejeon, Korea). PCR products were separated on a 2.5% agarose (Lonza, Basel, Switzerland) gel for electrophoresis.

### 2.3 Sequencing

To cross check the presence of KI BVPrP cDNA sequences in the targeted allele, the extracted gDNA was amplified using the primers listed in Table S1 (Set 2). PCR products were subjected to 1% agarose gel electrophoresis to check the sizes of the DNA fragments. Once the PCR products were properly formed, DNA from the rest of the PCR reaction was extracted using a PCR clean‐up kit (GeneAll Biotechnology, Seoul, Korea). The purified PCR products were sequenced (Cosmogenetech, Seoul, Korea) using the same primers used in the PCR reaction (Set 2 in Table S1).

### 2.4 Prion Infection in Mice

Five‐week‐old female C57BL/6N littermate WT, heterozygous KIBVPrP248, and homozygous KIBVPrP248 mice were anesthetized with isoflurane (Hana Pharm Co. LTD, Hwaseong, Korea) and then intracerebrally inoculated with 30 μL of 1% (w/v) RML prion inoculum, as described previously [24]. The mice were humanely euthanized when they exhibited multiple clinical signs of the terminal stage of prion disease. Tissue samples were collected, and the incubation period was recorded for each mouse. Separately, the brain slices of *Prnp*‐null mice (*Prnp* ^ZH3/ZH3^ [25], a kind gift from Dr. Adriano Aguzzi, University of Zurich, Switzerland) were also used as a negative control for the immunohistochemical analysis.

### 2.5 Western Blotting

The preparation of cultured cells and animal tissues for Western blotting was conducted as described elsewhere [24, 26]. Cultured cells in 100 mm dishes (Corning, New York, NY, USA) were lysed using 1 mL of cell lysis buffer containing 20 mM Tris–HCl (Sigma‐Aldrich), 150 mM NaCl (Sigma‐Aldrich), 0.5% Nonidet P‐40 substitute (Sigma‐Aldrich), and 0.5% sodium deoxycholate (Sigma‐Aldrich). Lysates were collected into 1.7 mL Eppendorf tubes and centrifuged at 7000 × g for 1 min to remove cell debris. The supernatant was saved at −80°C until use. For the preparation of mouse brain tissue homogenates (10% w/v), brains were homogenized in phosphate buffered saline (PBS; Thermo Fisher Scientific, Waltham, MA, USA) using an OMNI Bead Ruptor 24 (PerkinElmer, Shelton, CT, USA) with six ceramic beads (1.4 mm in diameter, Omni International, Kennesaw, GA, USA). Homogenates were centrifuged at 3000 × g for 5 min at 4°C, and the supernatant was saved at −80°C until use. Protein concentrations in the cell lysate and tissue homogenates were measured before use using a Pierce bicinchoninic acid protein assay kit (Thermo Fisher Scientific).

To detect PrP^C^ and ER stress/unfolded protein response (UPR)‐related proteins, 20 μg of total protein from cell lysates and 40 μg of total protein from brain homogenates were used. The protein samples were mixed with 4X sample loading buffer containing 8% SDS (Sigma‐Aldrich), 30% glycerol (Sigma‐Aldrich), 0.02% bromophenol blue (Sigma‐Aldrich), 250 mM Tris–HCl (pH 6.8; Sigma‐Aldrich), and 10% β‐mercaptoethanol (Sigma‐Aldrich) and then heated for 10 min at 95°C–100°C. Proteins, along with a GangNam STAIN prestained protein ladder (INTRON Biotechnology, Seongnam, Korea) for molecular weight reference, were separated on 15% SDS‐polyacrylamide gels at 60 V for 10 min, followed by 100 V for approximately 2 h. The proteins were transferred to polyvinylidene difluoride membranes (Merck Millipore, Burlington, MA, USA) at 300 mA for 1 h. The membranes were blocked with 5% skim milk (BD, Franklin Lakes, NJ, USA) or 3% bovine serum albumin (BSA; Sigma‐Aldrich) for 1 h at room temperature. Then, the membranes were incubated overnight with the primary antibody in Tris‐buffered saline with 0.05% Tween 20 (TBS‐T; Sigma‐Aldrich) at 4°C. The antibodies used in this study are listed in Table S2. After being washed with TBS‐T, the membranes were incubated with the secondary antibody in TBS‐T for 1 h at room temperature. The membranes were washed once again with TBS‐T and exposed to Amersham ECL Detection Reagents (Cytiva, Marlborough, MA, USA). Chemiluminescence from the detected proteins was visualized using a G:box Chem XR5 system (Syngene, Cambridge, UK). A densitometric analysis was performed using GeneTool software (Syngene). To show the level of the loading control, the membranes were incubated with anti‐β‐actin AC‐15 antibody and detected using the same procedure.

To detect PrP^Sc^ in mouse brains, 200 μg of protein from 10% (w/v) homogenates was incubated with an equal volume of 4% Sarkosyl (Fluka, Buchs, Switzerland) for 1 h at room temperature. Samples were then incubated with 20 μg/mL PK for 1 h at 37°C. Following PK digestion, 2 mM phenylmethylsulfonyl fluoride (MP Biomedicals, Santa Ana, CA, USA) was added to stop the reaction. The samples were centrifuged at 16,000 × g for 1 h at 4°C. The supernatant was discarded, and the pellet was suspended in 1X sample loading buffer, heated for 10 min at 95°C–100°C, and subjected to SDS‐polyacrylamide gel electrophoresis and Western blotting, as described above.

### 2.6 Cell Culture and Transfection

The rabbit kidney epithelial RK13 cell line (ATCC CCL‐37; ATCC, Manassas, VA, USA) was cultured at 37°C in a 5% CO_2_ incubator, as described previously [27]. The culture medium consisted of high glucose Dulbecco's Modified Eagle Medium (DMEM; Gibco, Grand Island, NY, USA), supplemented with 10% fetal bovine serum (Corning), 1% GlutaMAX (Gibco), and 1% penicillin/streptomycin (Gibco).

To establish RK13 cells expressing BVPrP, the following DNA constructs were used for transfection: pIRES‐puro3 (negative control; Clontech, Palo Alto, CA, USA), pIRES‐puro3_BVPrP255 (encoding the full 255 amino acids of BVPrP), and pIRES‐puro3_BVPrP248 (encoding 248 amino acids of BVPrP with partial C‐terminal signal sequence deletion). Information about the BVPrP DNA fragments is depicted in Figure S3A. To generate RK13 cells expressing green fluorescence protein (GFP) under the control of the N‐ and C‐terminal signal sequences of PrP, we used pIRES‐puro3 constructs containing either a complete (pIRESpuro3_GFP254) or incomplete (pIRESpuro3_GFP247) mouse PrP C‐terminal signal sequence (Figure S3B). RK13 cells were seeded in 6‐well plates (SPL Life Sciences, Pocheon, Korea) and transfected 24 h later with 2.5 μg of plasmid DNA and 7.5 μL of Lipofectamine 3000 (Thermo Fisher Scientific) according to the manufacturer's instructions. Then, the cell culture medium was replaced with fresh medium at 5 h post‐transfection. For transient transfection, cells were cultured for 48 h, and cell lysates were prepared for Western blot analysis. To compare transfection efficiency, 250 ng of pEGFP‐C2 plasmid (Clontech) was co‐transfected. GFP fluorescence was visualized using an Eclipse Ti‐E inverted fluorescence microscope (Nikon, Tokyo, Japan), and the fluorescent areas were quantified using ImageJ software (National Institutes of Health, Bethesda, MD, USA). To establish stable transfectants, the cells were cultured for 96 h in the replaced fresh medium, passed to 100 mm dishes (Corning), and maintained in culture medium with 2 μg/mL puromycin (Gibco). A negative control group transfected with the empty pIRES‐puro3 and a mock‐transfection group without DNA were included.

### 2.7 RNA Extraction, cDNA Synthesis, and RT‐qPCR

For total RNA extraction, confluent cells in a 6‐well plate were washed twice with Dulbecco's PBS (DPBS; Thermo Fisher Scientific), and 400 μL of TRIzol (Sigma‐Aldrich) per well was added for cell lysis. The lysate was transferred to a 1.7 mL tube, mixed thoroughly with 60 μL of chloroform (Sigma‐Aldrich), vortexed, and centrifuged at 12,000 × g for 15 min at 4°C. The aqueous phase was transferred to a new tube and mixed with an equal volume of isopropanol for precipitation. RNA was purified using an AccuPrep universal RNA extraction kit (Bioneer) and quantified using a Tecan Infinite 200 PRO spectrophotometer with a NanoQuant plate (Tecan, Männedorf, Switzerland). To extract RNA from mouse brain tissue, freshly extracted brains were homogenized using an OMNI Bead Ruptor 24 (PerkinElmer) in TRIzol, and the supernatant was processed as described above.

For cDNA synthesis, 2 μg of total RNA was used with a Takara PrimeScript 1st strand cDNA synthesis kit (Takara, Tokyo, Japan) in a 20 μL reaction. PCR confirmation of cDNA synthesis was performed using rabbit β‐actin primers (Table S1, Set 3) with AccuPower PCR premix (Bioneer), and the products were analyzed on a 2.5% agarose gel.

To analyze *Prnp* mRNA expression levels, SYBR Green–based RT‐qPCR was performed using a LightCycler 480 real‐time PCR system (Roche). Each 20‐μl reaction contained 10 μL of 2X Power SYBR Green PCR master mix (Applied Biosystems, Waltham, MA, USA), 0.5 μM primer sets (Table S1, Sets 3 and 4), and 1 μL of cDNA. Considering PCR efficiency, primers were designed to hybridize corresponding positions in the mouse and BV *Prnp* sequences and generate amplicons with similar sizes (Table S1, Set 4). The ΔΔCt method [28] was used to calculate relative gene expression.

### 2.8 Quantification of DNA Copy Number

To determine the copy number of plasmid DNA constructs integrated within the genome of stable transfectants of RK13 cells expressing the BVPrP gene, gDNA was extracted after the RNA extraction process by precipitation with 300 μL of absolute ethanol (Merck Millipore) per 1 mL of TRIzol sample [29]. After several washes with 70% ethanol, DNA was eluted in TE buffer (10 mM Tris–HCl, 1 mM EDTA, pH 8.0). Phenol extraction was performed to further purify the DNA. The DNA was precipitated by adding ammonium acetate (Sigma‐Aldrich) to a final concentration of 0.75 M and a 2.5‐fold excess volume of absolute ethanol, followed by centrifugation at 14,000 × g for 20 min at 4°C. The pellet was washed with 80% ethanol, eluted in TE buffer, and used for RT‐qPCR of BVPrP with the primers listed in Table S1 (Set 3). The copy number was determined by comparing the abundance of BVPrP amplicons produced from gDNA samples of RK13‐BVPrP255 and RK13‐BVPrP248 to a standard curve of those generated from a serially diluted, known amount of pIRESpuro3_BVPrP255 plasmid DNA.

### 2.9 PNGase F, PI‐PLC, MG132, and Bafilomycin A1 Treatment

To deglycosylate proteins in cell lysate, samples were denatured under a denaturing condition (0.5% SDS, 40 mM DTT) using 10X denaturing buffer (NEB, Ipswich, MA, USA) and heated for 10 min at 100°C [30]. After incubation with 500 U/μL of peptide: N‐glycosidase F (PNGase F; NEB) for 3 h at 37°C, the proteins were precipitated with absolute ethanol and resuspended in cell lysis buffer (20 mM Tris–HCl, 150 mM NaCl, 0.5% Nonidet P‐40 substitute, and 0.5% sodium deoxycholate) for Western blotting.

For the analysis of GPI‐anchoring, cultured cells were incubated in culture medium with 5 U/mL of phosphatidylinositol‐specific phospholipase C (PI‐PLC; Sigma‐Aldrich) for 3 h at 37°C. After incubation, the culture medium was collected, and the cells were lysed for further analysis. For proteasome inhibition, cells were incubated in culture medium with 10 μM MG132 (Sigma‐Aldrich) for 4 to 8 h. For lysosomal inhibition, cells were incubated in culture medium with 100 nM bafilomycin A1 (MedChem, Monmouth Junction, NJ, USA) for 4, 8, 12, and 24 h. The cell lysate was prepared as described above.

### 2.10 Immunofluorescence Staining

Double immunofluorescence staining was conducted by sequential incubation with two different staining combinations. The primary and secondary antibodies used for immunofluorescence staining are listed in Table S2. In the first experiment, cells were cultured on cover glasses coated with 0.2% gelatin (Sigma‐Aldrich) in 24‐well plates. The cells were washed with DPBS and incubated with Alexa Fluor 555–conjugated cholera toxin B (1:500 dilution, Thermo Fisher Scientific) for 30 min at 4°C. After being washed, the cells were fixed with 3.7% formalin (Duksan General Science, Seoul, Korea) for 10 min, permeabilized with 0.1% Triton X‐100 (Sigma‐Aldrich), and blocked with 2% BSA (Sigma‐Aldrich) for 30 min. The cells were incubated with anti‐PrP 8H4 antibody (1:500 dilution) for 1 h, followed by goat anti‐mouse IgG Alexa Fluor 488 for 1 h. In the second experiment, cells were fixed, permeabilized, and blocked as described above. Then, they were incubated with anti‐PrP 8H4 antibody (1:500 dilution) or anti‐Grp78 antibody (1:200 dilution) for 1 h, followed by goat anti‐mouse IgG Alexa Fluor 488 or goat anti‐rabbit IgG Alexa Fluor 555, respectively. After staining with 4′,6‐diamidino‐2‐phenylindole (DAPI; Thermo Fisher Scientific), the cells were mounted on slides and visualized using an Eclipse Ti‐E inverted fluorescence microscope (Nikon).

For GFP detection, the culture medium of RK13‐GFP254 and RK13‐GFP247 cells was replaced with phenol red–free DMEM (Gibco). GFP signals were directly observed in live cells using an Eclipse Ti‐E inverted fluorescence microscope (Nikon).

For tissue staining, 6‐μm thick coronal sections of mouse brains were deparaffinized, antigen‐retrieved by boiling them in 10 mM sodium citrate buffer (pH 6.0; Sigma‐Aldrich), and blocked with 5% normal goat serum (Vector Laboratories, Burlingame, CA, USA). Sections were incubated with anti‐PrP 6D11 (1:1500 dilution) and anti‐Grp78 (1:200 dilution) antibodies, followed by incubation with secondary antibodies conjugated with Alexa Fluor 647 and Alexa Fluor 488 and DAPI staining. Immunofluorescence images were captured using an Olympus FV3000 confocal microscope (Olympus, Tokyo, Japan). The colocalization of Grp78 and PrP was analyzed by measuring the pixel area of overlapping fluorescence signals using ImageJ software (National Institutes of Health).

### 2.11 mRNA Stability Assay

To determine mRNA stability in vitro, total RNA was extracted from RK13‐BVPrP255 and RK13‐BVPrP248 cells by the method described above and incubated in TE buffer at 37°C for various durations (0, 1, 2, 4, 10, and 24 h). At each time point, RNA was reverse‐transcribed into cDNA, and RT‐qPCR was performed using the BVPrP primers listed in Table S1 (Set 3) to measure the remaining *Prnp* mRNA levels. To evaluate intracellular mRNA stability, cells were incubated in culture medium with 5 μg/mL actinomycin D (Sigma‐Aldrich), a transcription inhibitor, for 2, 4, 8, and 14 h. At each time point, total RNA was extracted and reverse‐transcribed into cDNA, followed by RT‐qPCR.

### 2.12 In Vitro Transcription/Translation

To prepare templates for in vitro transcription/translation, DNA fragments encoding BVPrP255 and BVPrP248 were subcloned into the pcDNA3.1(+) vector (Thermo Fisher Scientific). The plasmids were linearized with SmaI (NEB), and in vitro transcription/translation was carried out using a wheat germ extract system (Promega, Madison, WI, USA). The reaction products were analyzed by Western blotting.

### 2.13 Immunoprecipitation

A 500‐μg protein sample in cell lysis buffer (20 mM Tris–HCl, 150 mM NaCl, 0.5% Nonidet P‐40 substitute, and 0.5% sodium deoxycholate) was incubated with washed Protein A/G PLUS‐Agarose (Santa Cruz Biotechnology, Dallas, TX, USA) with gentle rocking for 1 h at 4°C. The samples were centrifuged at 2500 × g for 5 min at 4°C, and the supernatant was transferred to a new tube. The protein sample was incubated with capture antibodies (anti‐PrP SAF32 or anti‐ubiquitin antibodies) with gentle rocking overnight at 4°C. Then, washed Protein A/G PLUS‐Agarose was added to the protein sample and incubated overnight with gentle rocking at 4°C. The protein sample was centrifuged at 2500 × g for 5 min at 4°C, and the pellet was washed with cell lysis buffer five times. The pellet was resuspended with 2X sample loading buffer, heated for 10 min at 95°C–100°C, and analyzed by Western blotting.

### 2.14 Immunohistochemistry

For PrP detection in the brain, deparaffinized 6‐μm thick coronal sections of mouse brain were antigen‐retrieved by boiling them in 10 mM sodium citrate buffer (pH 6.0) [31]. The sections were incubated in 3% hydrogen peroxide solution (Daejung Chemicals and Metals Co. Ltd., Siheung, Korea) for 5 min, permeabilized with 0.05% Triton X‐100 (Sigma‐Aldrich) for 10 min, and blocked with M.O.M. blocking reagent (Vector Laboratories) for 1 h. The sections were incubated with anti‐PrP 6D11 antibody (1:1500 dilution) for two days at 4°C, followed by biotinylated anti‐mouse IgG for 10 min. A VECTASTAIN Elite ABC‐HRP kit, peroxidase (Vector Laboratories), and Vector DAB substrate with nickel enhancement were used for detection. Nuclear fast red (Vector Laboratories) was used to counterstain in most slides, but not in a few to improve signal clarity.

### 2.15 Statistical Analysis

The incubation periods for prion‐inoculated WT and KIBVPrP248 mice were analyzed using a Kaplan–Meier survival analysis. Group comparisons were performed using two‐sided log‐rank tests in SPSS27 software (IBM, Armonk, NY, USA). Statistical differences in protein levels, mRNA expression, and mRNA stability between RK13‐BVPrP255 and RK13‐BVPrP248 were analyzed using Student's *t*‐test. A *p*‐value of 0.05 or less was considered statistically significant and marked as *; *p* ≤ 0.01 was marked as **; and *p* ≤ 0.001 was marked as ***.

## 3 Results

### 3.1 KIBVPrP248 Mice Exhibited Low PrP^C^ Expression and Resistance to RML Prion Infection

The KIBVPrP248 mouse line expressing amino acids 1 to 248 of BVPrP^C^ was generated. Because the complete protein‐coding sequences of mouse PrP^C^ are located in exon 3 of the *Prnp* gene, a targeting vector was designed to replace the open reading frame of endogenous PrP^C^ with that of BVPrP^C^ (Figure S1A).

Homologous recombination between the targeting vector and *Prnp* allele in the genome successfully yielded the targeted ES cell clones. One clone, 1A8, was selected for microinjection to blastocysts. Three male and five female heterozygous mice with the targeted allele derived from 1A8 were created. After the founders were established, breeding between them and with WT mice yielded offspring with heterozygous and, subsequently, homozygous KI alleles. To ensure the correct transmission of the KI allele, genotyping was performed using multiplex PCR (Figure S1B). Homozygous KIBVPrP248 mice yielded only a 206‐bp PCR product because it included a loxP site between the BVPrP cDNA sequence and the 3′ UTR. Heterozygous KIBVPrP248 mice produced both 167‐bp and 206‐bp PCR products. WT mice produced only the 167‐bp PCR product. These results suggest the successful introduction of the KIBVPrP248 allele.

To further verify that BVPrP was properly integrated into the genome without unintended mutations, the DNA sequence was determined using PCR products obtained from mouse gDNA. A BVPrP gene sequence different from that of the mouse *Prnp* gene was read from the gDNA of homozygous BVPrP KI mice, indicating the proper KI of the BVPrP gene (Figure S1C). Furthermore, the DNA sequence included only amino acids 1–248 of BVPrP, with a stop codon immediately following amino acid 248, indicating the lack of the 7 C‐terminal amino acids.

**FIGURE 1:**
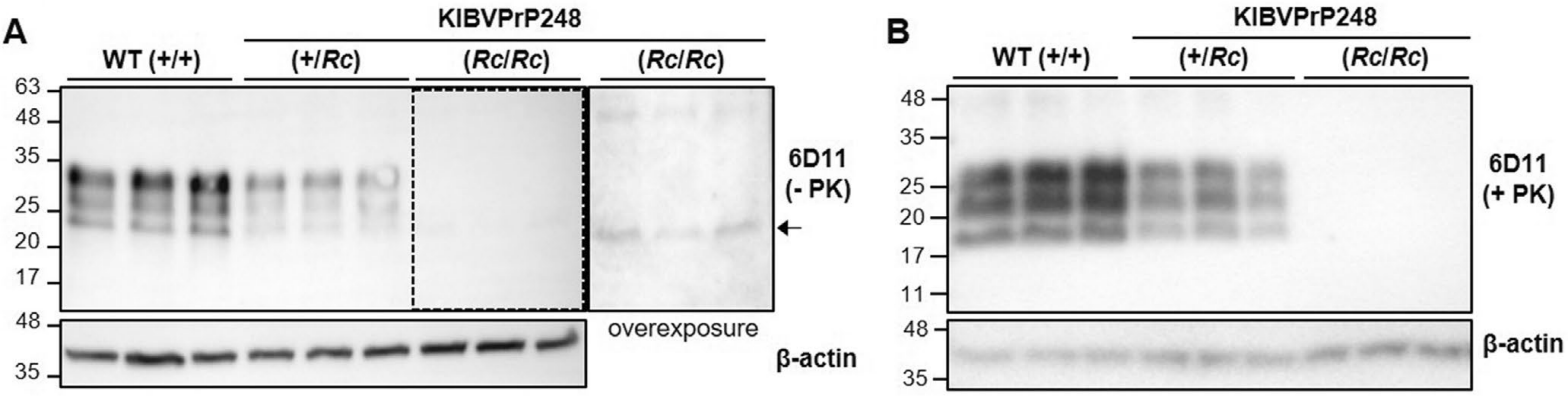
Western blot analysis of PrP^C^ and PrP^Sc^ in KIBVPrP248 mice. (A) Western blot analysis of PrP^C^ expression in the brains of littermate WT (+/+), heterozygous (+/*Rc*), and homozygous (*Rc*/*Rc*) KIBVPrP248 mice. The blot for homozygous KIBVPrP248 mouse samples was overexposed. The arrow indicates PrP^C^, likely in the unglycosylated form, that was barely expressed. (B) Western blot analysis of PK‐resistant PrP^Sc^ levels in the brains of RML prion–infected WT (+/+), heterozygous (+/*Rc*) and homozygous (*Rc*/*Rc*) KIBVPrP248 mice at the terminal stage of the disease. Western blots for β‐Actin represent the level of internal control for the experiment.

To assess PrP^C^ expression in the brains of BVPrP KI mice, Western blotting was performed. Heterozygous KIBVPrP248 mice showed reduced PrP^C^ expression compared with WT mice, and homozygous KIBVPrP248 mice expressed only a small amount of PrP^C^, likely in the unglycosylated form, and it was visible only upon overexposure (Figure 1A).

To investigate the susceptibility of KIBVPrP248 mice to prion infection, the mice were inoculated with RML prions, and the incubation period of the disease and PrP^Sc^ levels in the brain were assessed. The average incubation period for WT mice was 178.5 days, whereas for heterozygous KIBVPrP248 mice, it was extended to 380.5 days, approximately 2.1 times longer than for the WT mice (Table 1, Figure S2). In contrast, no homozygous KIBVPrP248 mice in the group showed any clinical onset of prion disease, and they all survived healthy for 452 days post‐infection until euthanasia. Western blotting of brain homogenates from these mice demonstrated that the heterozygous KIBVPrP248 mice showed lower PrP^Sc^ levels than the WT mice, and no PrP^Sc^ was detected in the homozygous KIBVPrP248 mice (Figure 1B). Because PrP^C^ expression is essential for prion propagation, the failure of prion infection in homozygous KIBVPrP248 mice is presumed to be due to their extremely low BVPrP expression. To further investigate the molecular basis for the reduced PrP^C^ expression in KIBVPrP248 mice, additional experiments were conducted using cellular models.

**TABLE 1:** Incubation periods in prion‐inoculated WT and KIBVPrP248 mice.

Groups	Prion strain	Mean incubation periods (days ± SEM)	*n*/*n*_0_	*p*
WT (+/+)	RML	178.5 ± 2.8	8/8	
KIBVPrP248 (+/*Rc*)	RML	380.5 ± 4.6	8/8	0.000087
KIBVPrP248 (*Rc*/*Rc*)	RML	> 452	0/7	0.000209

*Note:* Statistical analysis was performed using the log‐rank test.

Abbreviations: +/+, homozygote for wild type mouse *Prnp*; +/*Rc*, heterozygote of the mutant BV *Prnp* KI allele with wild type mouse *Prnp* background; *n*, number of prion‐ill mice; *n*_0_, total number of mice in the group; *Rc*/*Rc*, homozygote for the mutant BV *Prnp* KI allele; SEM, standard error of the mean.

### 3.2 RK13‐BVPrP248 Cells Expressed Unglycosylated PrP^C^ at a Low Level, and It Was Located in the ER

**FIGURE 2:**
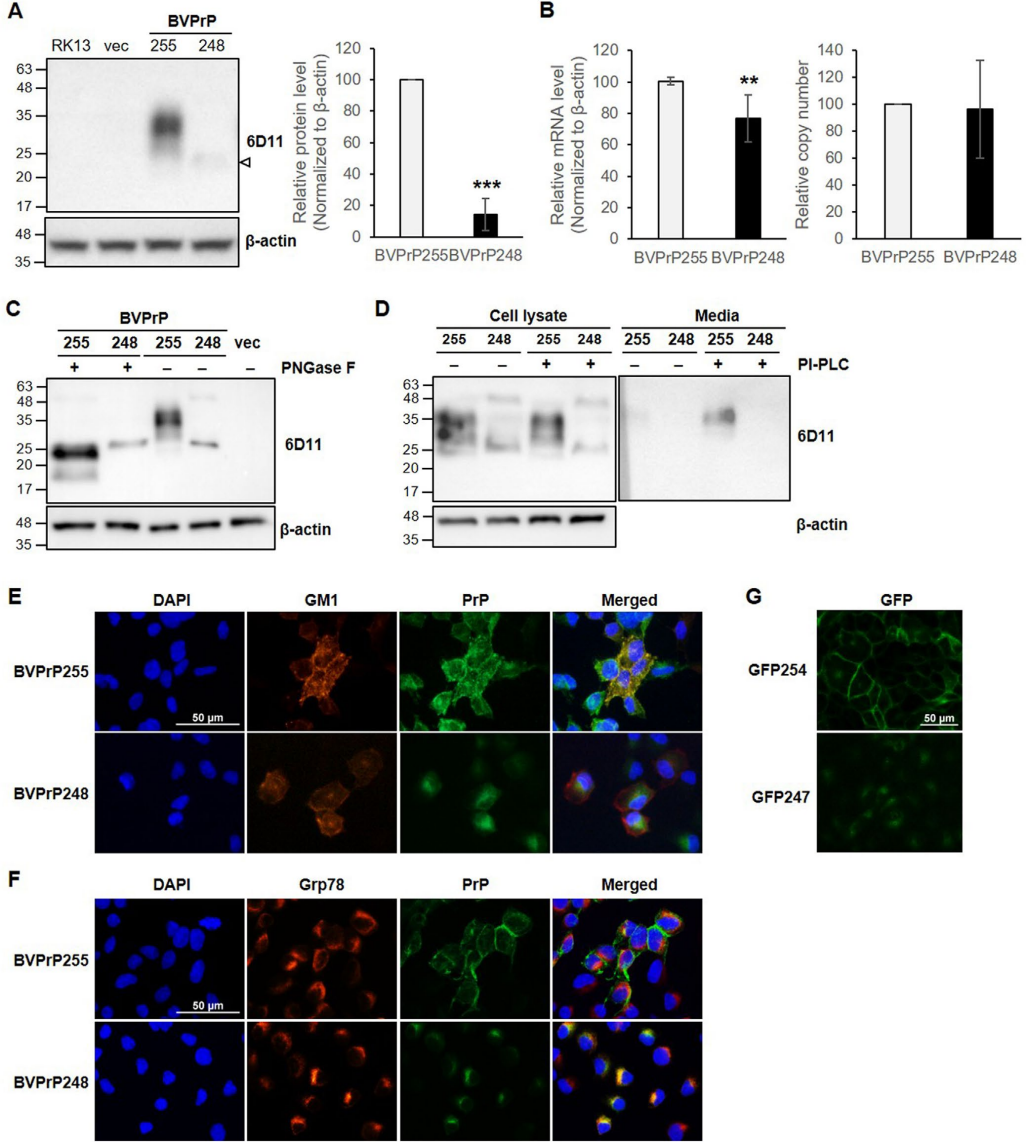
Characterization of RK13‐BVPrP248 cells. (A) Western blot analysis of BVPrP^C^ expression in RK13‐BVPrP255 and RK13‐BVPrP248 cells. RK13, mock‐transfected cells; vec, cells transfected with an empty vector. Densitometry of BVPrP^C^ levels normalized to β‐Actin (right panel, *n* = 4). (B) RT‐qPCR analysis of *Prnp* mRNA levels in RK13‐BVPrP255 and RK13‐BVPrP248 cells (*n* = 5). Relative copy number analysis via qPCR using gDNA (right panel, *n* = 5). (C) Western blot analysis of glycosylation status following PNGase F treatment. (D) Western blot analysis following PI‐PLC treatment to assess GPI‐anchoring in cell lysates and media. The media were collected from cultures of 1.5 to 1.9 × 10^6^ cells. (E, F) Double immunofluorescence staining of BVPrP and ganglioside GM1 (lipid raft marker) using cholera toxin B or Grp78 (ER marker) in RK13‐BVPrP255 and RK13‐BVPrP248 cells. Nuclei were stained with DAPI. Scale bar = 50 μm. (G) GFP detection in RK13‐GFP254 and RK13‐GFP247 cells. Scale bar = 50 μm. ***p < 0.001. **p < 0.01.

To investigate the cause of reduced BVPrP expression in the KIBVPrP248 mice, RK13 cell lines expressing either the full‐length BVPrP^C^, designated as BVPrP255, or the truncated BVPrP^C^, designated as BVPrP248 (mimicking the KIBVPrP248 mice), were established (Figure S3A). Western blot analyses determined the expression levels of PrP^C^. The RK13‐BVPrP255 cells expressed PrP^C^ with the typical glycosylation pattern, showing di‐, mono‐, and unglycosylated forms, whereas the RK13‐BVPrP248 cells displayed only a single band, presumably corresponding to the unglycosylated form, with a significantly lower expression level than the RK13‐BVPrP255 cells (Figure 2A). Quantification of the Western blot data revealed that BVPrP248 expression was approximately 14.3% of that of BVPrP255. These results suggest that the partial deletion of the C‐terminus in BVPrP affects protein expression because both RK13‐BVPrP248 cells and KIBVPrP248 mice exhibited reduced protein levels of PrP^C^.

To examine whether the reduced PrP^C^ protein expression in RK13‐BVPrP248 cells was due to transcriptional regulation, the *Prnp* mRNA levels were determined using RT‐qPCR. The *Prnp* mRNA level in RK13‐BVPrP248 cells was approximately 76.7% of that in the RK13‐BVPrP255 cells (Figure 2B). To examine whether the reduced mRNA level in RK13‐BVPrP248 cells was associated with a difference in DNA copy numbers caused by variations in transfection efficiency, qPCR was performed with gDNA from the cells. No statistically significant difference in copy numbers was shown between RK13‐BVPrP255 and RK13‐BVPrP248 cells (*p* = 0.833), indicating that the reduced level of mRNA transcripts in RK13‐BVPrP248 was not attributable to differences in copy numbers of the DNA constructs transfected (Figure 2B). However, the level of reduction in the *Prnp* mRNA transcripts does not fully explain the level of reduction in BVPrP248 expression at the protein level.

Normal PrP^C^ undergoes glycosylation and GPI‐anchoring during biosynthesis. To determine whether the PrP^C^ in RK13‐BVPrP248 cells underwent these post‐translational modifications, Western blotting was performed following deglycosylation with PNGase F or cleavage of GPI with PI‐PLC. After deglycosylation, BVPrP255 exhibited the unglycosylated form, confirming normal glycosylation, whereas the BVPrP248 showed only the unglycosylated band both before and after deglycosylation, indicating that it did not undergo glycosylation (Figure 2C). Interestingly, the migration of unglycosylated BVPrP248 was slightly retargeted in the Western blot, compared with that of the unglycosylated BVPrP255, suggesting the retention of a partial segment (amino acids 232–248) of the GPI‐SS in BVPrP248. To assess whether PrP^C^ was properly GPI‐anchored to the plasma membrane, Western blotting was performed with cell lysates and culture media after the cells were incubated with PI‐PLC. BVPrP255 tethered to the plasma membrane of the cells (and thus found mostly in the cell lysate) was released into the media in a greater amount after PI‐PLC treatment, suggesting that the GPI‐anchor for BVPrP255 had been cleaved, and PrP^C^ was released extracellularly, confirming proper GPI‐anchoring for BVPrP255 (Figure 2D). In contrast, BVPrP248 was detected in the cell lysate but not in the media both before and after PI‐PLC treatment, suggesting that BVPrP248 was not GPI‐anchored and remained intracellular.

To further investigate the subcellular localization of BVPrP248, double immunofluorescence staining was performed using cholera toxin B, a lipid raft marker that recognizes ganglioside GM1, and Grp78, an ER marker, along with PrP. In RK13‐BVPrP255 cells, BVPrP255 co‐localized with GM1 in the lipid rafts of the plasma membrane (Figure 2E). In RK13‐BVPrP248 cells, however, BVPrP248 was not co‐localized with GM1 in the plasma membrane. In contrast, BVPrP255 showed limited co‐localization with Grp78 in the ER of RK13‐BVPrP255 cells, representing the protein species in the normal biogenesis process of BVPrP255. Conversely, BVPrP248 exhibited strong co‐localization with Grp78 in the ER of RK13‐BVPrP248 cells, indicating its abnormal retention in the ER (Figure 2F). To confirm that the results observed in Figure 2E,F—where BVPrP255 was correctly localized to the lipid rafts and BVPrP248 was abnormally retained in the ER—were not due to the lack of glycosylation in BVPrP248, immunofluorescence was performed using cells expressing GFP, which does not undergo glycosylation, instead of the PrP core protein (Figure S3B). In RK13‐GFP254 cells, which expressed GFP with the complete N‐ and C‐terminal signal sequences of mouse PrP, GFP was localized to the cell membrane (Figure 2G). In contrast, in RK13‐GFP247 cells, which expressed GFP with complete N‐terminal and incomplete C‐terminal signal sequences, GFP was retained intracellularly. The results suggest that the change in intracellular location was due to the partial deletion of the C‐terminal signal sequence, not glycosylation depletion.

### 3.3 Reduced BVPrP248 Expression Is Primarily due to Proteasomal Degradation Rather Than Transcriptional or Translational Regulation

**FIGURE 3:**
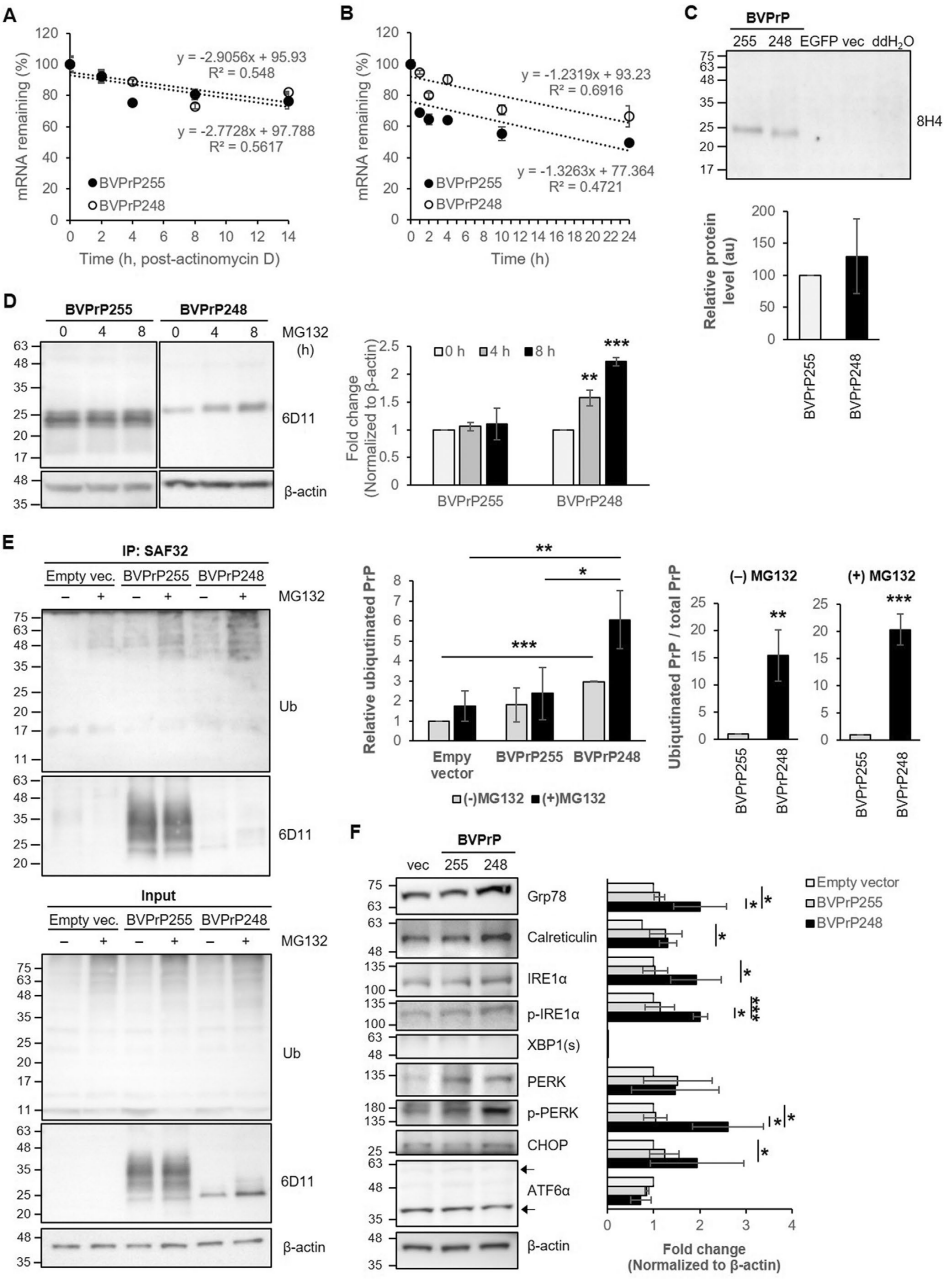
Mechanisms underlying BVPrP248 downregulation. (A) mRNA decay within RK13‐BVPrP255 and RK13‐BVPrP248 cells. Cells were treated with actinomycin D, and intracellular mRNA decay was measured at 2, 4, 8, and 14 h by RT‐qPCR. (B) In vitro mRNA stability. Total RNA isolated from RK13‐BVPrP255 and RK13‐BVPrP248 cells was left at 37°C for up to 24 h, followed by RT‐qPCR. (C) Western blot analysis of reaction products from a wheat germ extract–coupled in vitro transcription/translation system. EGFP was used as a positive control, and an empty vector (vec) and double distilled water (ddH₂O) were used as negative controls. The densitometry of the Western blots is shown in the bottom panel (*n* = 5). (D) Proteasomal degradation. The cell culture was incubated with a proteasome inhibitor (MG132). Total BVPrP levels were analyzed by Western blotting after PNGase F digestion. The densitometry of BVPrP levels was normalized to β‐Actin (right panel, *n* = 3). (E) Ubiquitination analysis. The empty vector–transfected RK13, RK13‐BVPrP255, and RK13‐BVPrP248 cells were incubated with or without MG132. Cell lysates were subjected to immunoprecipitation with an anti‐PrP SAF32 antibody, followed by Western blotting with an anti‐ubiquitin antibody. The densitometry of ubiquitinated PrP levels was analyzed with or without normalization to total PrP (right panels, *n* = 4). (F) Western blot analysis of ER stress and UPR‐related protein levels in RK13 cells transiently transfected with empty vector, BVPrP255, or BVPrP248, 48 h post‐transfection. The densitometry of marker proteins is shown in the right panel (*n* = 3). ***p < 0.001; **p < 0.01; *p < 0.05.

To determine which steps in protein biosynthesis primarily contribute to the reduced BVPrP248 protein level, we examined its mRNA stability, translation efficiency, and protein degradation. First, mRNA stability was assessed both in cells (Figure 3A) and in vitro (Figure 3B). RK13‐BVPrP255 and RK13‐BVPrP248 cells were incubated with actinomycin D, a transcription inhibitor, and mRNA was extracted at several time points (2, 4, 8, and 14 h post‐treatment). Following cDNA synthesis, RT‐qPCR was performed to measure mRNA decay within the cells. mRNA for both BVPrP255 and BVPrP248 decayed about 20% during 14 h (Figure 3A). The decay rate indicated by the degree of the slopes from a linear regression was nearly identical for both BVPrP255 and BVPrP248 mRNA over the time course, indicating no significant difference in mRNA decay between the groups. To assess mRNA stability in vitro, total RNA was extracted from RK13‐BVPrP255 and RK13‐BVPrP248 cells and left at 37°C for 1, 2, 4, 10, and 24 h. cDNA was synthesized at each time point, and the remaining *Prnp* mRNA levels were quantified by RT‐qPCR. Degradation of *Prnp* mRNA occurred rapidly during the first 1 h for BVPrP255 and 2 h for BVPrP248 to the 70%–80% level (Figure 3B). Then, mRNA levels gradually decreased to about 50% for BVPrP255 and about 65% for BVPrP248 at 24 h. Although the BVPrP255 mRNA degraded more rapidly during the early hours and remained less than BVPrP248 mRNA after 24 h, the decay rate in vitro was similar for both, as with the mRNA stability within cells. These data do not explain the reduced protein expression of BVPrP248.

Next, to assess translational regulation, the production of polypeptides for BVPrP255 and BVPrP248 was determined using in vitro translation reactions. Linearized plasmids with the BVPrP255 and BVPrP248 genes were subjected to wheat germ extract–based in vitro transcription/translation. The resultant protein levels, measured by Western blotting and densitometry, showed no significant difference between BVPrP255 and BVPrP248 in the relative protein levels (Figure 3C). The production of enhanced GFP, a positive control, was confirmed by detecting its fluorescence (Figure S4). The negative control reaction with the empty vector (without the gene insert) and the mock transfection control with water showed no in vitro translation products (Figures 3C and S4).

Finally, to assess protein turnover as an event of post‐translational regulation, both cell lines were incubated with MG132, a proteasome inhibitor, and cell lysates were collected at 0, 4, and 8 h. To compare the protein level of PrP^C^ in its unglycosylated state, Western blotting was performed after deglycosylation with PNGase F (Figure 3D). In RK13‐BVPrP255 cells, there was no significant change, with only a 1.1‐fold increase at 8 h. However, in RK13‐BVPrP248 cells, a 1.6 to 2.2‐fold increase was observed at 4 and 8 h, respectively, indicating that BVPrP248 was more susceptible to proteasomal degradation than BVPrP255. To further investigate whether the lysosomal pathway also contributes to protein turnover, both cell lines were incubated with bafilomycin A1, a lysosomal inhibitor. In RK13‐BVPrP255 cells, the protein accumulated progressively over 24 h, reaching approximately 3.7‐fold relative to the control at 0 h (Figure S5). In contrast, RK13‐BVPrP248 cells showed no definite time‐dependent increase following bafilomycin A1 treatment, indicating that the degradation of BVPrP248 was independent of the lysosomal pathway. Time‐dependent accumulation of LC3B, a marker of autophagosome–lysosome fusion, demonstrated effective inhibition of lysosome function by bafilomycin A1 in both cell lines. These results suggest that BVPrP248 was primarily degraded through the proteasomal pathway, with little to no involvement of lysosomal degradation.

For a protein to be targeted for proteasomal degradation, it must first be tagged with ubiquitin. To confirm whether BVPrP248 was more ubiquitinated, immunoprecipitation with an anti‐PrP antibody followed by Western blotting with an anti‐ubiquitin antibody was performed. The total ubiquitin levels (without immunoprecipitation) increased in RK13‐empty vector, RK13‐BVPrP255, and RK13‐BVPrP248 cells after proteasome inhibition with MG132 (Figure 3E, bottom panel). In the immunoprecipitated samples, ubiquitinated PrP levels in RK13‐BVPrP248 cells showed a tendency to increase without MG132 treatment (Figure 3E, upper panel and left graph). Upon MG132 treatment, ubiquitinated PrP levels were significantly elevated in RK13‐BVPrP248 cells compared to those of RK13‐empty vector and RK13‐BVPrP255 cells (Figure 3E, upper panel and left graph). Since total PrP expression differs between RK13‐BVPrP255 and RK13‐BVPrP248 cells, ubiquitinated PrP levels were normalized to total PrP for accurate comparison. The ubiquitinated PrP level was significantly higher for BVPrP248 compared to that of BVPrP255, even in the absence of MG132, showing approximately a 15.3‐fold increase (Figure 3E, right graphs). Following proteasome inhibition with MG132, the ubiquitinated PrP level for BVPrP248 increased even further, showing an approximately 20.3‐fold increase compared to that of BVPrP255 (Figure 3E, right graphs). These data suggest that BVPrP248 was very actively ubiquitinated, which in turn robustly directed it to proteasomal degradation, resulting in a low protein level in cultured cells.

### 3.4 BVPrP248 Activates the UPR in RK13‐BVPrP248 Cells

BVPrP248 was retained in the ER (Figure 2F) and subjected to degradation through ER‐associated degradation (ERAD) after protein production (Figure 3D,E). Therefore, we investigated whether BVPrP248 accumulation in the ER triggered ER stress and the UPR. The levels of Grp78 and calreticulin, which are general markers of ER stress, and IRE1α, PERK, and ATF6α, which are key UPR sensors, were examined by Western blotting, along with their downstream signaling molecules. When the proteasome was not inhibited by MG132, the levels of Grp78, calreticulin, and IRE1α were higher in RK13‐BVPrP248 cells than in RK13‐empty vector cells (Figure S6). When the proteasome was inhibited by MG132, the levels of total/phosphorylated IRE1α and PERK were slightly higher in RK13‐BVPrP248 cells than in RK13‐empty vector cells (Figure S6). However, such increases did not appear to be specific for BVPrP248 because there was no significant difference in the level of those markers between RK13‐BVPrP248 and RK13‐BVPrP255 cells. The cleaved fragments of ATF6α (36 and 60 kDa fragments in rabbit cells [32]) remained unchanged among the cell lines (Figure S6). These results suggest that BVPrP248 expression and following accumulation in stable transfectants were insufficient to lead to full activation of the UPR pathways.

Next, we performed transient transfection to drive more active expression of BVPrP248 and analyzed UPR‐related proteins because it is possible that the stable cell line had already adapted to ER stress due to chronic expression of BVPrP248 and degradation by proteasomes, leading to a lack of significant changes in the expression of UPR‐related proteins. In the cells transiently transfected with pIRES‐puro3_BVPrP248, Grp78 was markedly upregulated (Figure 3F). Both phosphorylated IRE1α and phosphorylated PERK, which did not show BVPrP248‐specific increases in the stable cell line, were apparently increased, suggesting activation of these UPR pathways. Calreticulin, total IRE1α, and CHOP levels were higher in RK13‐BVPrP248 cells compared to those in RK13‐empty vector cells, but these differences were not observed when compared to those in BVPrP255. ATF6α levels remained unchanged, consistent with observations in the stable cell line. Transfection efficiency was constant across the groups transfected with different BVPrP plasmid constructs as fluorescence from the co‐transfected internal expression control GFP was not different (Figure S7). Taken together, these results suggest that acute overexpression of BVPrP248 induced ER stress and preferentially activated the IRE1α and PERK branches of UPR.

### 3.5 BVPrP248 Is Also Degraded by ER‐Mediated Proteasomal Degradation and Activates the UPR in KIBVPrP248 Mice

**FIGURE 4:**
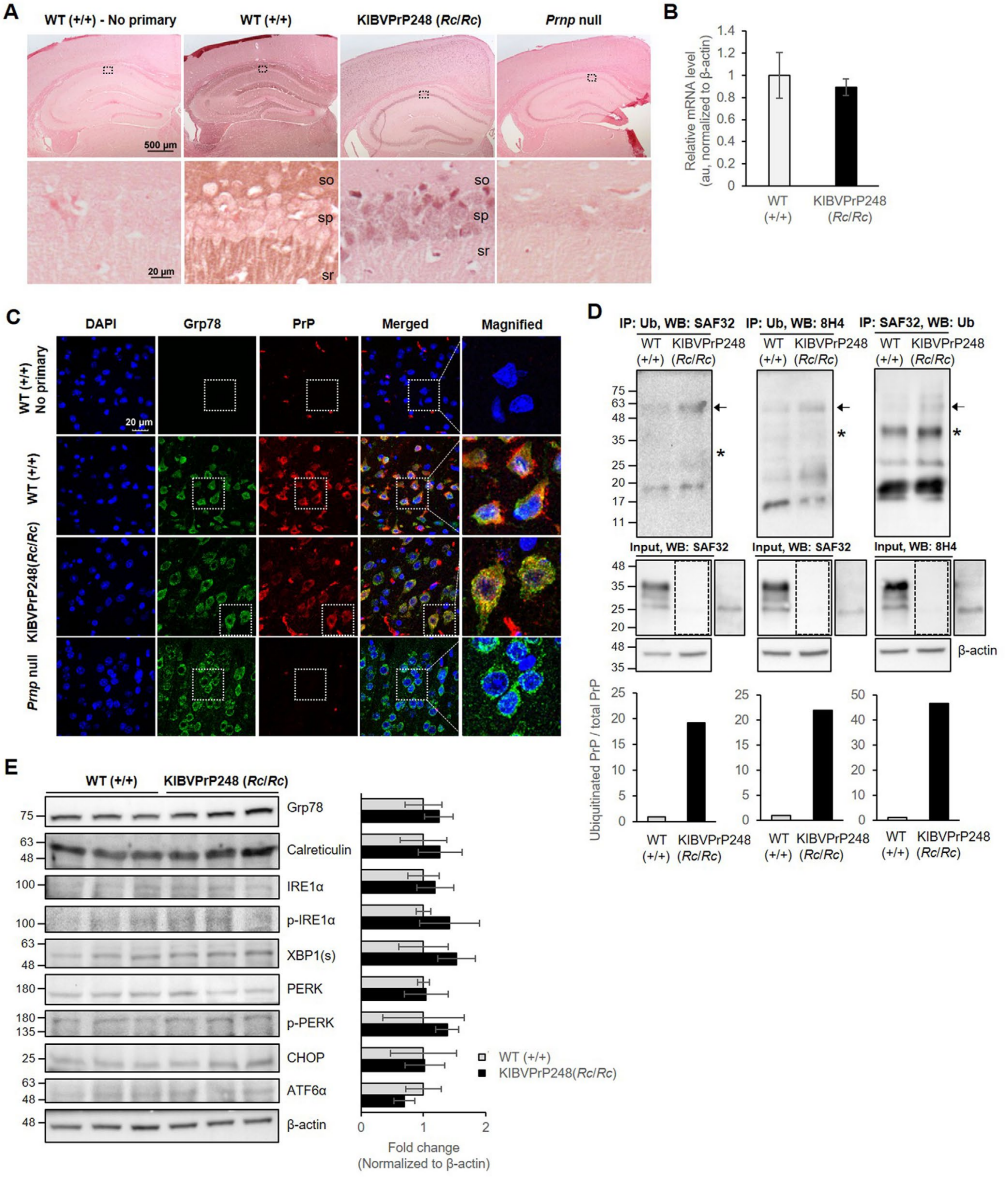
ER‐associated degradation of BVPrP248 and UPR responses in KIBVPrP248 mice. (A) Immunohistochemistry of brain tissue from littermate WT, KIBVPrP248 (*Rc*/*Rc*), and *Prnp* null mice. Sections were counterstained with Nuclear fast red. Scale bar = 500 μm (upper panel) and 20 μm (lower panel). The CA1 region of the hippocampus (box with discontinuous line) was magnified to compare the pattern of expressed BVPrP^C^ (lower panels). so, stratum oriens; sp., stratum pyramidale; sr, stratum radiatum. (B) *Prnp* mRNA expression levels in the brains of KIBVPrP248 (*Rc*/*Rc*) mice, as analyzed by RT‐qPCR. (C) Double immunofluorescence staining of Grp78 and PrP in the cortex of mouse brain. The co‐localization was visualized in merged images. Magnified images were obtained from the area marked with discontinuous boxes. Scale bar = 20 μm. (D) Ubiquitination of BVPrP in KIBVPrP248 (*Rc*/*Rc*) mice. Mouse brain homogenate was immunoprecipitated with anti‐PrP SAF32 antibody or anti‐ubiquitin antibody, and detected with anti‐ubiquitin antibody or anti‐PrP (SAF32 or 8H4) antibody, respectively. Arrows, tetra‐ubiquitinated PrP. Asterisks, mono‐ or di‐ubiquitinated PrP. The predicted molecular size of these bands was calculated based on addition of approximately 8.5–34 kDa from mono‐ to tetra‐ubiquitins to approximately 22.9–24.7 kDa from WT PrP and BVPrP. The expression of BVPrP as an unglycosylated form in KIBVPrP248 was shown in the overexposed input blots. The densitometry of tetra‐ubiquitinated PrP levels normalized to total PrP (bottom panel). (E) Western blot analysis of UPR‐related protein levels in WT and KIBVPrP248 (*Rc*/*Rc*) mouse brains. The densitometry of marker proteins (right panel, *n* = 3).

In RK13‐BVPrP248 cells, the low abundance of PrP^C^ was largely due to post‐translational proteasomal degradation (Figure 3D,E). To confirm whether the same mechanism was facilitated in KIBVPrP248 mice, we conducted experiments similar to those performed with cultured cells. First, immunohistochemistry on mouse brain tissue showed that the level of PrP^C^ expression was reduced in KIBVPrP248 mice, compared with WT mice that expressed PrP^C^ widely throughout the brain (Figure 4A, upper panel), corresponding to the results of Western blotting in Figure 2A. For instance, PrP^C^ expression was barely found in the stratum oriens and stratum radiatum of the CA1 hippocampal region in KIBVPrP248 mice, compared with the corresponding regions in WT mice, where PrP^C^ was detected rather abundantly in a diffused manner (Figure 4A, lower panel). In KIBVPrP248 mice, PrP^C^ was predominantly localized in the stratum pyramidale of the CA1 hippocampal region, where nuclei are densely packed (Figure 4A, lower panel). Notably, although BVPrP248 was lightly stained due to its low expression, the perinuclear localization of BVPrP248 in the stratum pyramidale of the CA1 region was clearly visible in the brain slice without counterstaining (Figure S8). Contrary to PrP^C^ at the protein level, an RT‐qPCR analysis of *Prnp* mRNA levels in mouse brain tissue revealed no significant difference between WT and KIBVPrP248 mice, indicating that the reduced protein levels were not due to changes in mRNA abundance in vivo (Figure 4B).

To assess whether PrP was similarly retained in the ER of brain cells in KIBVPrP248 mice, as in RK13‐BVPrP248 cells, double immunofluorescence staining was performed. In WT mice, although a certain level of PrP signals overlapped with Grp78, representing PrP species that undergo normal biogenesis processes, a substantial portion of PrP was not co‐localized with Grp78 and presented on the plasma membrane (Figure 4C). In contrast, in KIBVPrP248 mice, PrP was localized within the ER, mostly co‐localizing with Grp78, but not on the cell membrane. The *Prnp* null mouse brain slices used as a negative control showed no PrP staining.

To determine whether BVPrP248 is ubiquitinated as a substrate for proteasomal degradation in KIBVPrP248 mice, immunoprecipitation was performed using anti‐PrP or anti‐ubiquitin antibodies, followed by Western blotting with the opposite antibody. The molecular weight of unglycosylated WT PrP was estimated to be approximately 22.9 kDa, whereas BVPrP248, which retains the partial C‐terminal signal sequence instead of undergoing GPI‐anchor modification, was predicted to have a molecular weight of 24.7 kDa. Upon conjugation with four ubiquitin residues, which are properly recognized by proteasomes, the molecular size of ubiquitinated BVPrP was expected to increase to approximately 56.9 kDa and 58.7 kDa, respectively. These poly‐ubiquitinated bands appeared more prominently in KIBVPrP248 mice than in WT mice (Figure 4D, arrows), confirming that abnormal BVPrP248 is also targeted for proteasomal degradation in vivo. The ubiquitinated PrP levels in KIBVPrP248 mice were markedly elevated, showing approximately 20 to 47‐fold increases when normalized with total PrP levels (Figure 4D). In addition to those bands representing ubiquitinated BVPrP with four ubiquitin residues, the molecular size of some bands corresponds to that of mono‐ or di‐ubiquitinated BVPrP (Figure 4D, asterisks), while the tri‐ubiquitinated BVPrP band was not obviously detected. Other unmarked bands might represent PrP species from which ubiquitin was removed after the immunoprecipitation step or internally cleaved PrP fragments with various ubiquitination states.

To address the involvement of the lysosomal pathway in the reduced expression of BVPrP248 observed in mice, we examined the levels of Rab7, a marker for late endosomes and lysosomes, and LC3B, a marker for autophagosome‐lysosome fusion, using mouse brain samples. The Rab7 and LC3B levels were not significantly different between WT and KIBVPrP248 mice, suggesting that the lysosomal pathway was not activated in KIBVPrP248 mice (Figure S9). These results indicate that, as in the cultured cell model, it was unlikely that BVPrP248 was degraded through the lysosomal pathway in the mouse brain.

Because the IRE1α and PERK pathways were activated in RK13 cells transiently expressing BVPrP248, Western blotting was performed to examine whether the same pathways were activated in KIBVPrP248 mice. These mice did not show a significant difference in the level of marker proteins for ER stress or UPR compared to WT mice (Figure 4E), similar to the results demonstrated in stable transfectants of RK13‐BVPrP248 cells. These findings indicate that no obvious ER stress and UPR were induced in KIBVPrP248 mice.

## 4 Discussion

This study was initiated by the accidental observation that PrP^C^ expression was significantly reduced in KI mice harboring a BVPrP sequence with an unintended deletion of seven C‐terminal amino acids in the GPI‐SS (Figure 1A). These mice were not susceptible to infection with RML prions (Figure 1B, Table 1). However, this resistance was not attributable to a species barrier against RML prions because BVPrP^C^ has been reported to serve as a universal substrate for various prion strains [33]. Specifically, WT BVPrP^C^ KI mice generated in our group were susceptible when challenged with RML infection (unpublished data). In our effort to explain the low expression of BVPrP^C^ in KIBVPrP248 mice, we found that the partial deletion of the GPI‐SS C‐terminal sequence in PrP did not affect mRNA stability or translational efficiency, suggesting that the early stages of protein biosynthesis remained intact (Figures 2A,B and 3A–C). However, the BVPrP248 protein was subsequently degraded by the proteasome, likely due to the retention of the GPI‐SS, instead of its replacement by a GPI‐anchor, leading to its recognition as a misfolded protein (Figure 3D). Previous studies have shown that PrP retaining the GPI‐SS, generated in cells lacking GPI‐transamidation activity, is degraded through ERAD [34]. The presence of the GPI‐SS in BVPrP248 was further supported by a Western blot analysis of PNGase F–treated samples, where BVPrP248 exhibited retarded migration in sodium dodecylsulfate polyacrylamide gel electrophoresis, compared with BVPrP255 (Figure 2C). The consistent 2.6 kDa size difference between BVPrP248 and BVPrP255 suggests that BVPrP248 retained an uncleaved GPI‐SS, preventing efficient GPI‐anchoring and leading to degradation.

The GPI‐SS is composed of a hydrophilic spacer sequence of 8 to 12 amino acids following the GPI attachment site, and that is followed by a hydrophobic region of 8 to 20 amino acids [35, 36]. Although the amino acid sequence of PrP varies slightly between species, the GPI‐SS of PrP generally comprises 11 hydrophilic spacers and 14 hydrophobic residues [37]. Studies have shown that the deletion of the entire GPI‐SS from PrP results in protein secretion outside the cell, whereas the partial deletion of the hydrophobic region of PrP results in the protein being detected in both the cell lysate and media, indicating partial secretion. In the case of the other GPI‐anchoring protein, VSG117, partial GPI‐anchoring was observed when eight of the 15 hydrophobic amino acids in the GPI‐SS were deleted from the C‐terminus [38]. In this study, we confirmed that BVPrP248, with the deletion of seven of the 14 hydrophobic amino acids, did not undergo GPI‐anchoring and was not secreted extracellularly. The failure of BVPrP248 to be GPI‐anchored while retaining its GPI‐SS can be attributed to a reduction in hydrophobicity, which is essential for efficient GPI anchoring. The hydrophobicity of the C‐terminal region must be maintained within an optimal range for effective recognition by GPI transamidase; excessive or insufficient hydrophobicity disrupts this process [39, 40]. Previous studies have demonstrated a correlation between decreased hydrophobicity and reduced GPI anchoring efficiency [38, 40]. In the case of BVPrP248, the seven deleted C‐terminal amino acids (LIFLIVG) are highly hydrophobic, as indicated by their Kyte‐Doolittle hydrophobicity score [41]. The loss of those residues significantly decreases the overall hydrophobicity of the GPI‐SS, likely impairing its recognition by GPI transamidase and thereby impairing GPI‐anchoring.

The hydrophobicity of the GPI‐SS has been reported to influence the ER translocation pathway, which in turn affects the likelihood of interaction with oligosaccharyltransferase complexes. In the M232R PrP mutant, reduced hydrophobicity of the GPI‐SS redirected the protein to the hSND‐2‐dependent ER translocation pathway, resulting in decreased glycosylation [12]. Similarly, the reduced glycosylation observed in BVPrP248 suggests that the loss of hydrophobicity might have disrupted normal ER translocation, reducing the interaction with oligosaccharyltransferase. Furthermore, the lack of glycosylation in BVPrP248 could be due to the failure of its C‐terminus to anchor to the ER membrane. Previous studies suggested that N‐glycosylation does not occur when the C‐terminal signal sequence is completely deleted or when the protein is anchored to the ER membrane via the N‐terminus [14, 15]. In contrast, glycosylation occurs when the C‐terminal transmembrane domain or a non‐PrP GPI‐SS is present, indicating that C‐terminal anchoring in the ER membrane is essential for N‐glycosylation [14, 22]. Given that N‐glycosylation efficiency depends on proper ER membrane anchoring, it is likely that the loss of hydrophobicity in BVPrP248 contributed to both its GPI‐anchoring failure and glycosylation defects.

When misfolded proteins accumulate in the ER, exceeding its folding capacity, ER stress occurs, activating the UPR [42]. The UPR is activated by three sensors: IRE1α, PERK, and ATF6, and it regulates genes involved in protein folding, protein degradation, attenuation of protein synthesis, and ER chaperones [43]. In this study, ER stress was induced only when cultured cells were driven to overexpress BVPrP248 (Figure 3F). Notably, Grp78, phosphorylated IRE1α, and phosphorylated PERK were more prominently upregulated in the transiently transfected RK13‐BVPrP248 cells compared to the stable cell line (Figures 3F and S7). Grp78 is a key regulator that binds to UPR sensors to keep them inactive and activates the UPR when it binds to accumulating misfolded proteins in the ER [44]. Grp78 also mediates the translocation of misfolded proteins for proteasomal degradation [45]. Similar to the report that Grp78 associates with mutant PrP that retains an uncleaved GPI‐SS [46], it is likely that Grp78 binds to BVPrP248, which also retains a truncated, uncleaved GPI‐SS. Furthermore, the IRE1α and PERK pathways of UPR were activated in this cell culture model (Figure 3F). Because the IRE1α/XBP1 pathway promotes ERAD and protein folding [47], the data from this study suggest that BVPrP248 degradation occurs as a protective response to mitigate ER stress. However, activation of the PERK/CHOP pathway suggests that ER stress was not fully alleviated by protective mechanisms and had reached a level that sufficiently triggers pro‐apoptotic signaling [42]. Collectively, the partial deletion of the GPI‐SS reflected in RK13‐BVPrP248 cells was sufficient to potentially induce ER stress and UPR activation. However, these molecular events did not occur in stable transfectants of RK13‐BVPrP248 cells and KIBVPrP248 mice (Figures S6 and 4E). This phenomenon might be attributed to the adaptation of cells and animals to the condition in which aberrant BVPrP248 was produced at a low level, presumably maintained by proteasomal degradation, despite chronic expression. Thus, the same deletion of the GPI‐SS did not lead to acute pathological conditions, suggesting that C‐terminally truncated PrP might be well tolerated in vivo.

Although this study provides important mechanistic insights, several caveats could be considered. First, the cells used in this study were rabbit kidney cells that were commonly used to study exogenous PrP expression because they do not express endogenous PrP^C^ [48, 49]. Although the overall results from cultured cells corresponded to those shown in animals, the cellular response to ectopic expression of aberrant PrP^C^ might be different in rabbit non‐neuronal cells compared with mouse neuronal cells that highly express PrP^C^. This difference in cell type and species might have contributed to the variations observed in UPR–associated proteins, such as the presentation of spliced XBP1 fragments and the different cleavage pattern of ATF6α (Figures 3F, S7 and 4E). Second, unlike KIBVPrP248 mice, the RK13 cell model was an overexpression system. Despite the concerns for uncontrolled BVPrP gene overexpression between individual transfectants, the copy number of the BVPrP gene integrated into the gDNA in stable cell lines and the transfection efficiency in transiently transfected cells did not differ between the RK13‐BVPrP255 and RK13‐BVPrP248 cells. Thus, our comparative experiments between these internally controlled cell models are valid. Third, the partial C‐terminal deletion examined in this study does not represent a naturally occurring prion disease mutation. Thus, it is difficult for this study to interpret the mechanistic pathogenesis of genetic Creutzfeldt‐Jakob disease–associated GPI‐SS mutations [12].

This study suggests a potential strategy for intervening in prion diseases by sequestering PrP^C^ intracellularly, thereby preventing prion propagation. Interestingly, KIBVPrP248 mice remained disease‐free over 450 days after prion inoculation (Figure S2, Table 1), suggesting that targeting the C‐terminal amino acid residues in GPI‐SS could be a promising strategy for treating prion diseases. Recently, a strategy employing cytosine and adenine base editing systems to introduce a premature stop codon at the R37 position was attempted, resulting in a 50% reduction of PrP levels in the mouse brain and a 52% extension in survival [50]. In addition, antisense oligonucleotide‐based therapies have been shown to extend the lifespan of prion‐infected mice by 24%–46% [51] and a preventive approach using CRISPR/Cas9 technology to introduce the protective G127 polymorphism has also been proposed [52]. From the perspective of these therapeutic strategies, this study suggests that modification of DNA sequences for GPI‐SS at the gene level to suppress PrP^C^ expression could be a novel tool to block infection permanently. Moreover, the lack of physiological abnormalities, ER stress, and UPR activation in KIBVPrP248 mice makes this strategy worthy of further exploration as a potential therapeutic approach.

In summary, our findings demonstrate that the deletion of seven C‐terminal amino acids impairs GPI anchoring, reduces glycosylation, and promotes ER retention, leading to proteasomal degradation and the reduction of PrP^C^ expression. These results highlight the critical role of GPI‐SS hydrophobicity in PrP^C^ maturation and provide mechanistic insights into the role of GPI‐SS in PrP^C^ biosynthesis.

## Author Contributions

The study was conceived and designed by Miryeong Yoo and Chongsuk Ryou. The study was supervised by Chongsuk Ryou. Miryeong Yoo, Jieun Kim, Sunyeong Cha, Min Young Lee, Yeon Jeong Hwang, Woo‐Ri Ko, Taeeun Kim, A‐ran Kim, and Trang H.T. Trinh performed experiments and collected data. Miryeong Yoo and Sungeun Lee analyzed data. Chongsuk Ryou, Yong‐Pil Cheon, and Young‐Mi Kim guided experiments and supported experimental materials and research funding. The first draft of the manuscript was written by Miryeong Yoo, and all authors commented on subsequent versions of the manuscript. The manuscript was finalized by Chongsuk Ryou. All authors read and approved the final manuscript.

## Conflicts of Interest

The authors declare no conflicts of interest.

## Supporting information


**Data S1:** Supporting Information.

## Data Availability

The data that support the findings of this study are available upon request from the corresponding author. The data are not publicly available due to privacy or ethical restrictions.
