# Supplementary material for: Partial Deletion of the Carboxyl‐Terminal Signal Sequence of the Cellular Prion Protein Alters Protein Expression via Endoplasmic Reticulum–Associated Degradation
Source: FASEB J. 2025 Sep 4;39(17):e71016. doi: 10.1096/fj.202501227RR (PMC12410294; doi:10.1096/fj.202501227RR)
Supplement: Supplementary file 1 — Data S1: Supporting Information. [file FSB2-39-e71016-s001.docx]

**Supplementary data**

**Partial deletion of the carboxyl-terminal signal sequence of the cellular prion protein alters protein expression *via* endoplasmic reticulum–associated degradation**

**Authors**

Miryeong Yoo^1^, Sungeun Lee^1^, Jieun Kim^1^, Sunyeong Cha^2^, Min Young Lee^2^, Yeon Jeong Hwang^2^, Woo-Ri Ko^2^, Taeeun Kim^2^, A-ran Kim^1^, Trang H.T. Trinh^1,3^, Young-Mi Kim^1^, Yong-Pil Cheon^2^, Chongsuk Ryou^1*^

**ORCID ID**

Miryeong Yoo: https://orcid.org/0009-0001-0949-1593

Yong-Pil Cheon: https://orcid.org/0000-0002-8497-9257

Chongsuk Ryou: https://orcid.org/0000-0001-8363-1059

**Affiliations**

^1^ Department of Pharmacy, College of Pharmacy, and Institute of Pharmaceutical Science & Technology, Hanyang University ERICA, Ansan, Republic of Korea

^2^ Division of Developmental Biology and Physiology, Department of Biotechnology, Institute for Basic Sciences, Sungshin University, Seoul, Republic of Korea

^3^ Department of Pharmacy, East Asia University of Technology, Hanoi, Vietnam

* **Corresponding author**: Chongsuk Ryou, Tel: +82-31-400-5811; Fax: +82-31-400-5958; email: [cryou2@hanyang.ac.kr](mailto:cryou2@hanyang.ac.kr); address: 55 Hanyangdaehak-ro, Ansan, Gyeonggi-do, 15588, Republic of Korea

**Supplemental figures**

**
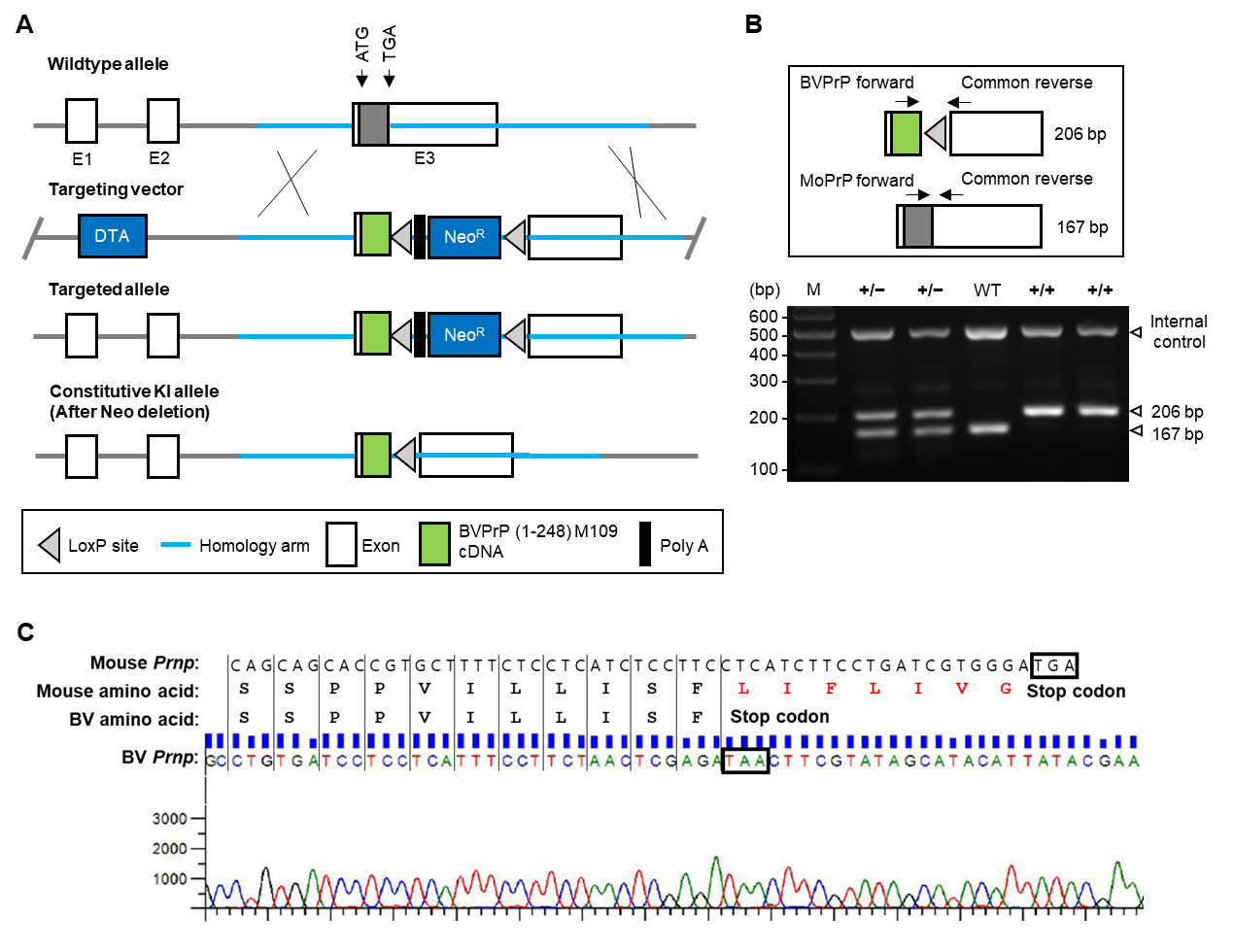
**

**Figure S1. Generation of KIBVPrP248 mice.** (A) Schematic representation of the targeting strategy used to generate KIBVPrP248 mice. DTA, diphtheria toxin A gene; Neo, neomycin resistance gene. (B) Genotyping of KIBVPrP248 mice by multiplex PCR. Homozygous (*Rc*/*Rc*) KIBVPrP248 mice yielded a 206-bp PCR product. Heterozygous (+/*Rc*) KIBVPrP248 mice produced both 167-bp (WT) and 206-bp (KI) products. Littermate WT mice produced only the 167-bp product. The locations of the primers are indicated in the box. (C) Comparison of DNA and amino acid sequences of the BVPrP gene from the BVPrP KI allele with those of the mouse PrP gene. Only the C-terminal portion of the PrP sequences is shown.

**
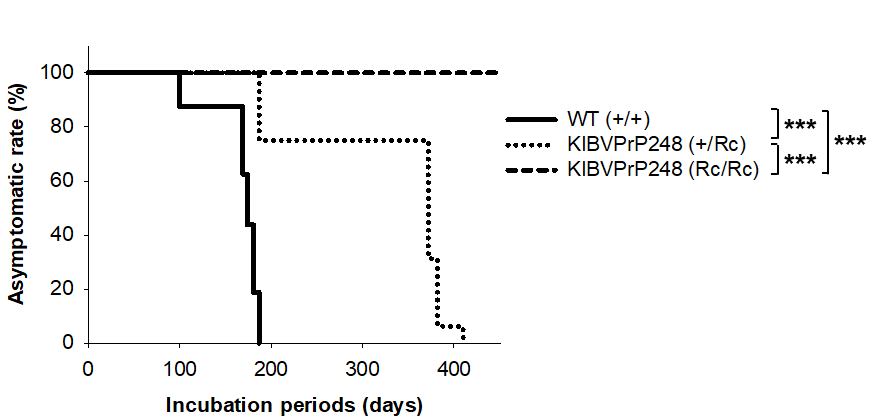
**

**Figure S2. Survival curves of prion-inoculated WT and KIBVPrP248 mice.** The survival of littermate WT (n=8) versus heterozygous KIBVPrP248 (+/*Rc*) (n=8) mice, littermate WT (n=8) versus homozygous KIBVPrP248 (*Rc*/*Rc*) (n=7) mice, and heterozygous KIBVPrP248 (+/*Rc*) (n=8) versus homozygous KIBVPrP248 (*Rc*/*Rc*) (n=7) mice was compared. ***, *p* < 0.001.

**
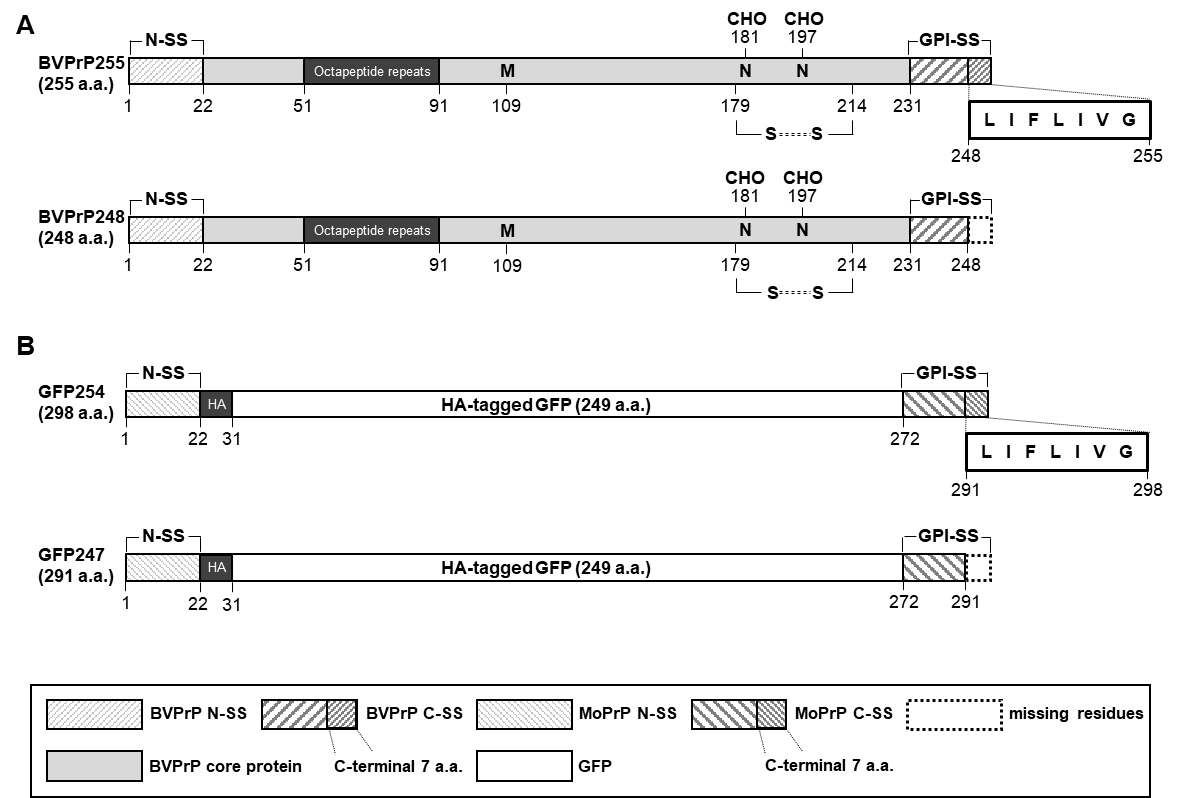
**

**Figure S3. Schematic representation of BVPrP and GFP constructs used to establish stable RK13 cell lines**. (A) Composition of the BVPrP255 and BVPrP248 constructs. (B) Composition of the GFP254 and GFP247 constructs. These constructs consist of the N-terminal signal sequence of mouse PrP, an HA tag, GFP, and the amino acid sequence spanning residues 228 to 254 (or 247) of mouse PrP, including the full or partial GPI anchoring signal sequences. N-SS, N-terminal signal sequence; CHO, glycosylation site; S=S, disulfide bonds; GPI-SS, GPI-anchoring signal sequence.

**
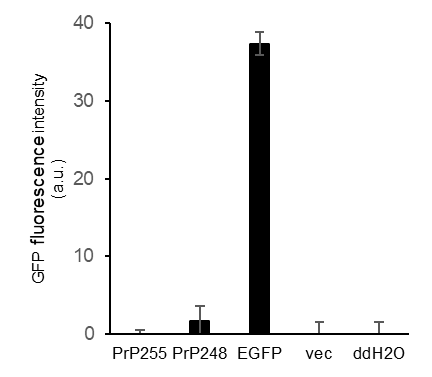
**

**Figure S4. GFP fluorescence intensity of wheat germ *in vitro* transcription/translation reaction products.** GFP fluorescence intensity was measured using a Tecan microplate reader at excitation 485 nm and emission 535 nm. EGFP was used as a positive control. A high fluorescence intensity of EGFP indicates that the *in vitro* transcription/translation reaction proceeded properly.

**
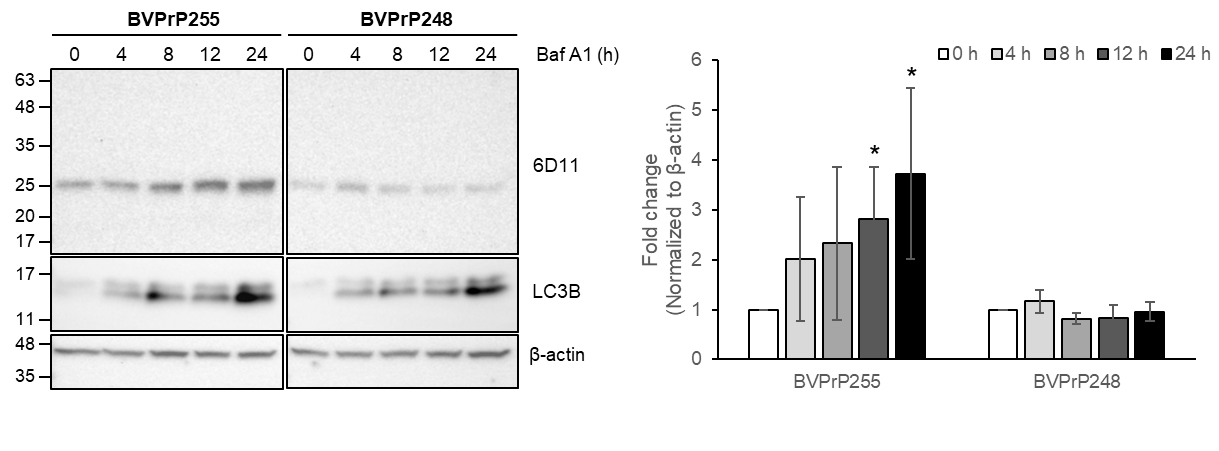
**

**Figure S5. PrP degradation by the lysosomal pathway in cultured cells.** Western blot analysis of total BVPrP levels in RK13-BVPrP255 and RK13-BVPrP248 cells treated with bafilomycin A1. Cell lysates were treated with PNGase F. An increase in LC3B confirms inhibition of the lysosomal pathway by Bafilomycin A1. The densitometry of BVPrP levels normalized to β-actin (right panel, n=3). *, *p* < 0.05.

**
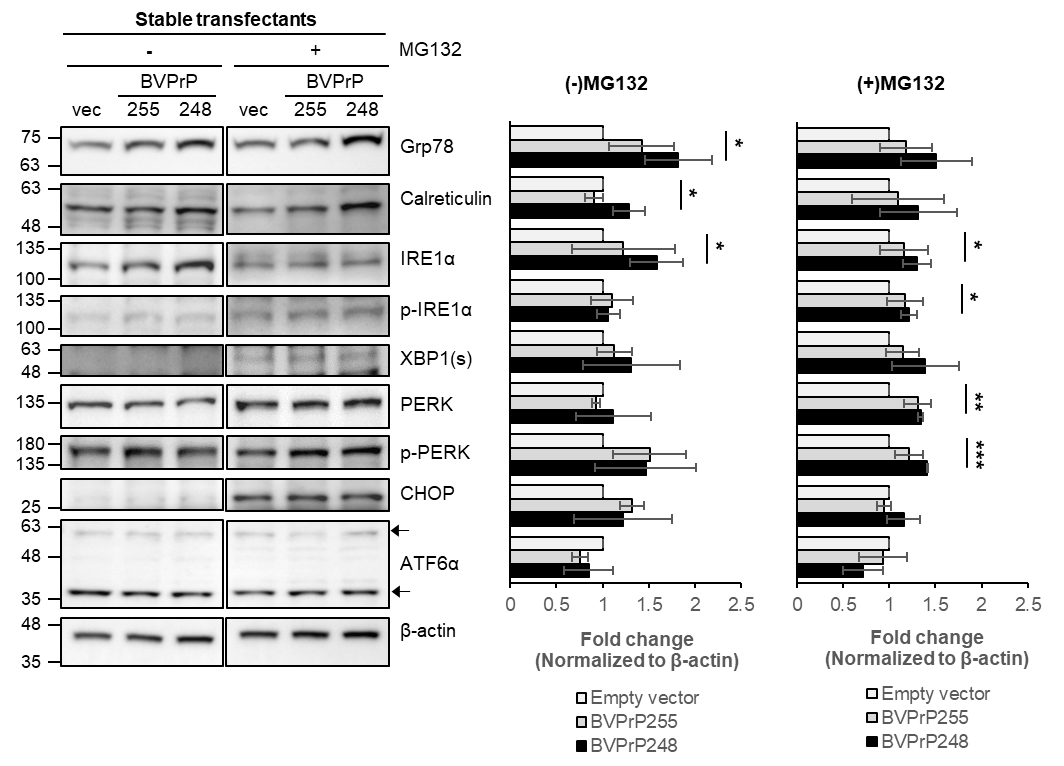
**

**Figure S6. ER stress and UPR-related protein levels in stable transfectants of RK13-BVPrP255 and RK13-BVPrP248 cells.** The cells were incubated with or without MG132. Cell lysates were analyzed by Western blotting for ER stress and UPR-related proteins. In representative blots, the level of some proteins such as Grp78 and calreticulin were increased in RK13-BVPrP248 cells. However, densitometry of multiple Western blots did not show significant increases specific for BVPrP248 (right panels, n=3). ***, *p* < 0.001. **, *p* < 0.01. *, *p* < 0.05. Arrows, two distinct fragments of cleaved ATF6α found in rabbit cells.

**
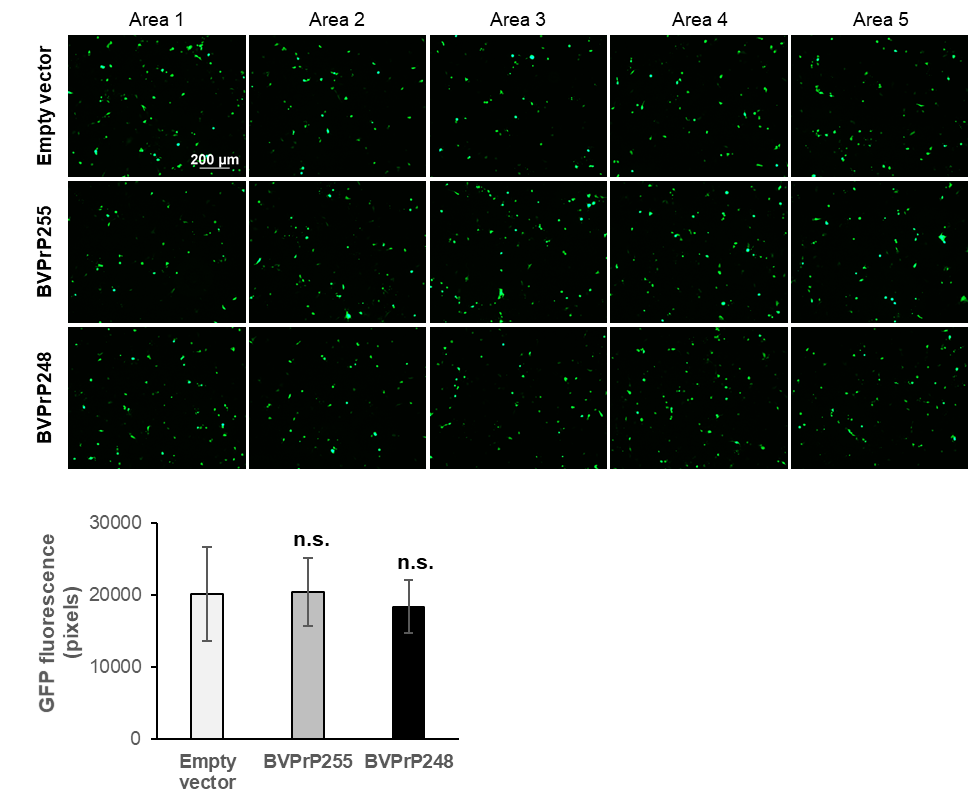
**

**Figure S7. Comparison of transfection efficiency.** GFP fluorescence of transiently co-transfected RK13-empty vector, RK13-BVPrP255, or RK13-BVPrP248 cells with pEGFP-C2 plasmid. Five randomly selected fields are shown. GFP fluorescence were quantified using ImageJ (n=5). Scale bar = 200 µm.

**
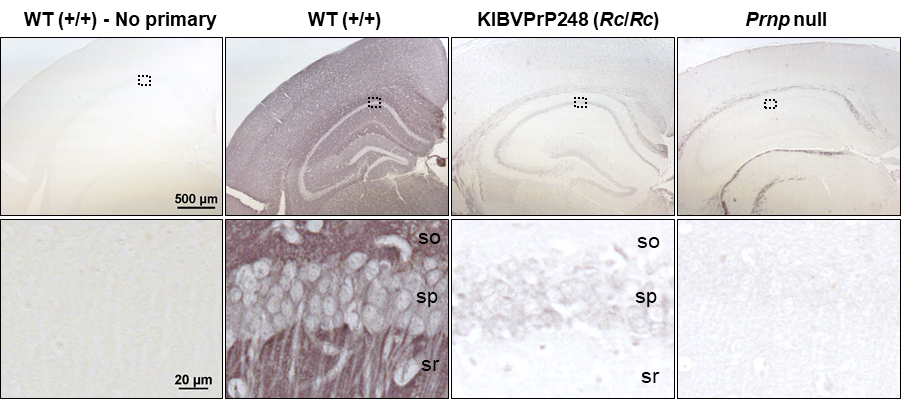
**

**Figure S8. Immunohistochemistry of PrP in littermate WT, KIBVPrP248 (*Rc*/*Rc*), and *Prnp* null mouse brains.** Staining of PrP signal was carried out by the same method used to generate the data shown in Fig. 4A, except for omitting counterstaining step to better visualize PrP signal. The CA1 region of the hippocampus (box with discontinuous line) was magnified to compare the pattern of expressed PrP^C^ (lower panels). so, stratum oriens; sp, stratum pyramidale; sr, stratum radiatum. Scale bar = 500 µm (upper panel) and 20 µm (lower panel).

**
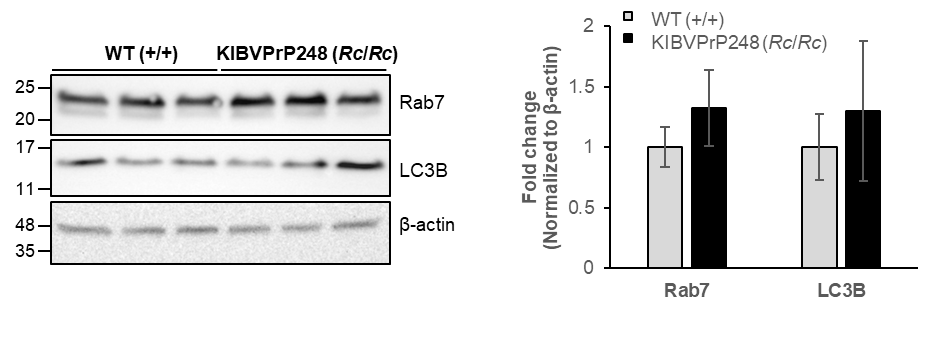
**

**Figure S9. Activation of lysosomal degradation in mouse brains.** Western blot analysis of Rab7 and LC3B in WT and KIBVPrP248 (*Rc*/*Rc*) mice. The densitometry of Rab7 and LC3B levels normalized to β-actin (right panel, n=3).

**Supplemental Tables**

Table S1. List of PCR primers used in this study

| **Primer set** | **Application** | **Primer name** | **Sequence (5’→3’)** | **Remarks** |
| --- | --- | --- | --- | --- |
| Set 1 | Genotyping for WT and KIBVPrP248 mice | Mo_PrP F1 | CGACGGGAGAAGATCCAGCA | - |
|  |  | BV_PrP F1 | GTGATCCTCCTCATTTCCTTCTAA | - |
|  |  | Common R | GAAAGAGCTACAGGTGGATAACCC | - |
|  |  | Mo_GAPDH F1 | CTGGTAACTCCGCCTTTGCG | Internal control |
|  |  | Mo_GAPDH R1 | GAATACGCATTATGCCCGAGG |  |
| Set 2 | Sequencing for KIBVPrP248 mice | BV_PrP F2 | CAGCCCTGGAGGCAACCG | - |
|  |  | Common R | GAAAGAGCTACAGGTGGATAACCC | - |
| Set 3 | RT-qPCR for stably transfected RK13 cells | BV_PrP F3 | GGAGGACCGCTACTACCGTGAA | - |
|  |  | BV_PrP R1 | CACGCGC TCCATCATCTTGACG | - |
|  |  | Rb_β-actin F | CGTGCGGGACATCAAGGAGA | Internal control |
|  |  | Rb_β-actin R | CACGACTCCATGCCCAGGAA |  |
| Set 4 | RT-qPCR  for WT and KIBVPrP248 mice | Mo_PrP F2 | GCCTGGAGGGTGGAACACT | - |
|  |  | Mo_PrP R1 | GTTGACCCCAACCGCCACC | - |
|  |  | BV_PrP F2 | CAGCCCTGGAGGCAACCG | - |
|  |  | BV_PrP R2 | GGCTTGTTCCACTGAT TGTG | - |
|  |  | Mo_β-actin F2 | GAGCACAGCTTCTTTGCAGCTCCT | Internal control |
|  |  | Mo_β-actin R2 | GGTAGGATACCTCTCTTGCTCTG |  |

Mo, mouse; BV, bank vole; Rb, rabbit; F, forward; R, reverse.

Table S2. List of antibodies used in this study

| **Antibody** | **Host** | **Clonality** | **Dilution** | **Manufacturer** | **Product No.** |
| --- | --- | --- | --- | --- | --- |
| PrP (6D11) | mouse | monoclonal | 1:30000 | BioLegend (San Diego, CA, USA) | 808003 |
| β-actin | mouse | monoclonal | 1:5000 | Santa Cruz Biotechnology (Dallas, TX, USA) | sc-69879 |
| PrP (8H4) | mouse | monoclonal | 1:1000 | Sigma-Aldrich (St. Louis, MO, USA) | P0110 |
| PrP (SAF32) | mouse | monoclonal | 1:1000 | Cayman Chemical (Ann Arbor, MI, USA) | A03202 |
| Ubiquitin | rabbit | polyclonal | 1:500 | Merck Millipore (Burlington, MA, USA) | 07-375 |
| Grp78 | rabbit | polyclonal | 1:1000 | Abcam (Cambridge, UK) | ab21685 |
| Calreticulin | rabbit | polyclonal | 1:1000 | Thermo Fisher Scientific (Waltham, MA, USA) | PA3-900 |
| IRE1α | rabbit | polyclonal | 1:1000 | Novus Biologicals (Centennial, CO, USA) | NB100-2324SS |
| p-IRE1α | rabbit | polyclonal | 1:1000 | Abcam | ab48187 |
| XBP1(s) | rabbit | polyclonal | 1:1000 | Novus Biologicals | NBP1-77681SS |
| PERK | rabbit | polyclonal | 1:1000 | Santa Cruz Biotechnology | sc-13073 |
| p-PERK T982 | rabbit | polyclonal | 1:1000 | ABclonal (Woburn, MA, USA) | AP0886 |
| ATF4 | rabbit | monoclonal | 1:1000 | Cell Signaling Technology (Danvers, MA, USA) | 11815 |
| eIF2α | rabbit | polyclonal | 1:1000 | Cell Signaling Technology | 9722 |
| p-eIF2α | rabbit | polyclonal | 1:1000 | Cell Signaling Technology | 9721 |
| CHOP | rabbit | polyclonal | 1:1000 | Novus Biologicals | NBP2-13172 |
| ATF6 | mouse | monoclonal | 1:1000 | Novus Biologicals | NBP1-40256SS |
| Rab7 | rabbit | monoclonal | 1:1000 | Cell Signaling Technology | 9367 |
| LC3B | rabbit | monoclonal | 1:2000 | Cell Signaling Technology | 3868 |
| Goat anti-mouse IgG (H+L) secondary antibody, HRP | goat | polyclonal | 1:10000 | Thermo Fisher Scientific | 31430 |
| Goat anti-rabbit IgG (H+L) secondary antibody, HRP | goat | polyclonal | 1:10000 | Thermo Fisher Scientific | 31460 |
| Goat anti-mouse IgG (H+L) cross-adsorbed secondary antibody, Alexa Fluor™ 488 | goat | polyclonal | 1:1000 | Thermo Fisher Scientific | A-11001 |
| Goat anti-mouse IgG H&L (Alexa Fluor® 647) | goat | polyclonal | 1:1000 | Abcam | ab150115 |
| Goat anti-rabbit IgG (H+L) cross-adsorbed secondary antibody, Alexa Fluor™ 488 | goat | polyclonal | 1:1000 | Thermo Fisher Scientific | A-11008 |
